# Molecular Phylogenetic Evaluation of Classification and Scenarios of Character Evolution in Calcareous Sponges (Porifera, Class Calcarea)

**DOI:** 10.1371/journal.pone.0033417

**Published:** 2012-03-27

**Authors:** Oliver Voigt, Eilika Wülfing, Gert Wörheide

**Affiliations:** 1 Department of Earth and Environmental Sciences, Ludwig-Maximilians-Universität München, München, Germany; 2 GeoBio-Center LMU, Ludwig-Maximilians-Universität München, München, Germany; 3 Bayerische Staatssammlung für Paläontologie und Geologie, München, Germany; Biodiversity Insitute of Ontario - University of Guelph, Canada

## Abstract

Calcareous sponges (Phylum Porifera, Class Calcarea) are known to be taxonomically difficult. Previous molecular studies have revealed many discrepancies between classically recognized taxa and the observed relationships at the order, family and genus levels; these inconsistencies question underlying hypotheses regarding the evolution of certain morphological characters. Therefore, we extended the available taxa and character set by sequencing the complete small subunit (SSU) rDNA and the almost complete large subunit (LSU) rDNA of additional key species and complemented this dataset by substantially increasing the length of available LSU sequences. Phylogenetic analyses provided new hypotheses about the relationships of Calcarea and about the evolution of certain morphological characters. We tested our phylogeny against competing phylogenetic hypotheses presented by previous classification systems. Our data reject the current order-level classification by again finding non-monophyletic Leucosolenida, Clathrinida and Murrayonida. In the subclass Calcinea, we recovered a clade that includes all species with a cortex, which is largely consistent with the previously proposed order Leucettida. Other orders that had been rejected in the current system were not found, but could not be rejected in our tests either. We found several additional families and genera polyphyletic: the families Leucascidae and Leucaltidae and the genus *Leucetta* in Calcinea, and in Calcaronea the family Amphoriscidae and the genus *Ute*. Our phylogeny also provided support for the vaguely suspected close relationship of several members of Grantiidae with giantortical diactines to members of Heteropiidae. Similarly, our analyses revealed several unexpected affinities, such as a sister group relationship between *Leucettusa* (Leucaltidae) and Leucettidae and between *Leucascandra* (Jenkinidae) and *Sycon carteri* (Sycettidae). According to our results, the taxonomy of Calcarea is in desperate need of a thorough revision, which cannot be achieved by considering morphology alone or relying on a taxon sampling based on the current classification below the subclass level.

## Introduction

Calcarea Bowerbank, 1864 is one of the four currently recognized classes of Porifera [1], [2]. Its relationship to the other main sponge classes, i.e., Demospongiae Sollas, 1885, Hexactinellida Schmidt, 1870 and Homoscleromorpha Bergquist, 1978, has long been unclear, especially because molecular analyses have questioned the monophyly of Porifera. However, the hypothesis of sponge paraphyly was often poorly supported by the data or was hampered by the lack of representatives of what we today refer to as the four sponge classes [3]. More recently, phylogenomic studies found high support for sponge monophyly and a sister group relationship between Calcarea and Homoscleromorpha [3], [4].

Calcareous sponges comprise approximately 675 accepted extant species [2]; therefore, this class is considerably less diverse than for example, the most species-rich class, Demospongiae, which contains approximately 7.000 recognized species [2]. Calcarean species are exclusively marine animals. Most species occur at shallow depths and only few species are known from the deep sea (for an overview see e.g., [5]). In contrast to other sponges, which have siliceous spicules, all calcareous sponges build calcite spicules, which constitute a synapomorphy of the group [6]. Within Calcarea, the relationships are little understood. However, this small group of sponges has long been of interest to zoologists because of the variety of simple and more complex organization forms found in extant species, and also because of their apparent beauty caused by the occasionally geometrical arrangement of their skeletons. Among the first to be fascinated by the organizational diversity in Calcarea was Haeckel, who for this reason focussed on this group to establish ‘a natural system’ of Calcarea in his monograph ‘Die Kalkschwämme’ [7]–[9] to promote the emerging ideas of Darwinism. Since the days of Haeckel, the most important characters used in the taxonomy of Calcarea are the organization of the aquiferous system and skeletal features.

### The aquiferous system of Calcarea

**Figure 1:**
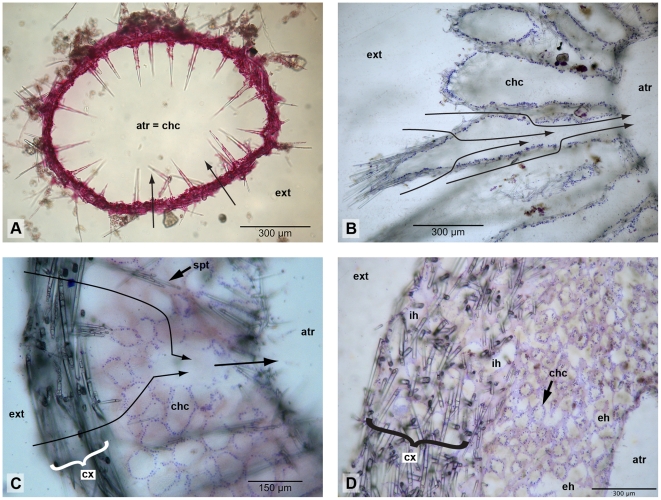
Different organizations of the aquiferous system in Calcarea. A: asconoid (*Soleneiscus radovani*); B: syconoid (*Sycon coronatum*, from Helgoland, Germany); C: sylleibid (*Grantiopsis cylindrica*); D: leuconoid (*Leucettusa* sp. 1). Thin arrows illustrate the direction of water flow in A, B and C. atr = atrium; chc = choanocyte chambers; cx = cortex; eh = exhalant channel; ext: exterior of the sponge; ih = inhalant channel; spt = spicule tract of modified triactines.

Sponges are filter feeders that create a unidirectional water current through their bodies by the beating central flagella of specialized cells, the choanocytes. The microvilli collar of a choanocyte captures food particles, which are taken up by the cell. Traditionally, four different types of aquiferous systems can be readily distinguished in Calcarea: the asconoid, syconoid, sylleibid and leuconoid grades of organisation. Recently, a fifth type was described, the solenoid system [10]. In asconoid sponges, all internal cavities of the sponge are lined by choanocytes (Fig. 1, A). Such sponges are also called homocoel. All other organization forms of the aquiferous system are heterocoel, i.e., some parts of the internal cavities are lined by pinacocytes, a cell type that also covers the external surfaces of sponges. In syconoid Calcarea, choanocytes are organized in elongated chambers, which are radially arranged around the atrium; water enters the sponge via inhalant canals and the choanocyte chambers via pores. The choanocyte chamber opens to the central cavity (the atrium), which is lined by pinacocytes (Fig. 1, B). Sylleibid sponges have radially arranged choanocyte chambers, which do not open directly into the atrium. Instead, several choanocyte chambers open into a cavity lined by pinacocytes, which itself has an opening to the atrium (Fig. 1, C). The most complex organization of the aquiferous system is the leuconoid grade. Here, water enters the sponge through a system of inhalant canals leading to numerous, approximately spherical choanocyte chambers. These chambers open to exhalant canals, through which the water reaches the atrium (Fig. 1, D). In some leuconoid species, the atrium is strongly reduced. Recently, Cavalcanti and Klautau [10] introduced the new term solenoid to describe the organisation of the aquiferous system found in the genus *Leucascus* and to stress the differences with syconoid sponges. The solenoid aquiferous system is characterized by choanocytes, which are restricted to anastomosed tubes, and pinacocytes, which line the atrial cavity. Accordingly, solenoid sponges are also heterocoel. Calcarea is the only sponge class in which all of these different types of aquiferous systems are present.

### Skeletal arrangement in Calcarea

Spicule morphology in Calcarea is rather limited compared to the occasionally very elaborate siliceous spicules of other sponges (e.g., Hexactinellida, [11]). In extant Calcarea, spicules can be categorized in just three types, i.e., diactines, triactines and tetractines, depending on the number of growing rays of the spicule. Pentactines were also reported but are only known from one species, *Sycon pentactinale* [12]. Modifications of these spicule types can occur. However, in most cases, the arrangement of spicules in the skeleton (in combination with the nature of the aquiferous system) has been considered more phylogenetically informative for higher classifications than the form of the spicules itself.

**Figure 2:**
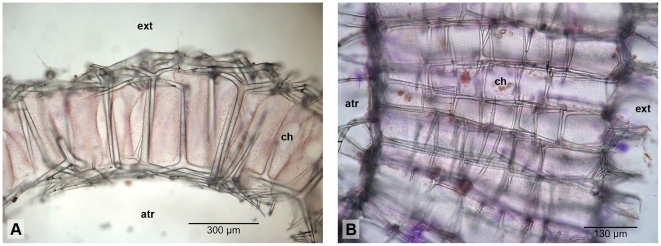
Organization of choanoskeleton. A: inarticulated (*Sycettusa* aff. *hastifera*); B: articulated (*Grantessa* sp. GW974).

In the simplest calcareans, the skeleton consists of only one layer of spicules, which supports the pinacoderm on the outer side, and the choanoderm on the inner side of the sponge (e.g., Fig. 1, A). More complex skeletons can be divided into an atrial skeleton, which supports the wall of the atrial cavity, and a choanoskeleton, which supports the choanosome. In sponges with thin walls, the choanosome is only supported by unpaired actines of subatrial spicules and, depending on the sponge, also by actines of subcortical or cortical spicules (Fig. 2, A). Such a skeletal organization is termed inarticulated choanoskeleton. In contrast, articulated choanoskeletons are built from several, roughly parallel rows of similar spicules, usually sagittal triactines with their unpaired actines pointing to the outside of the sponge (Fig. 2, B). With such an arrangement, sponges can build thick walls. The choanosome of thick walled sponges can also be supported by numerous spicules, without apparent order, or by spicular tracts of modified triactines. Reinforced skeletons can be formed by fused (occasionally modified) spicules or by an aspicular calcite mass. A tangential layer of spicules that covers the external surface of the sponge is called the cortex. It can be thin, formed by a single layer of spicules, or thick, and occasionally primarily sustain the sponge wall (Fig. 1, C & D).

#### Classification of Calcarea

Manuel [13] offers a short overview of the history of the classification of Calcarea. In the current classification, Calcarea is subdivided into two subclasses, Calcinea and Calcaronea [2], [14], [15]. This concept was originally based on a cellular character, the position of the nucleus within the choanocytes, first proposed to separate some asconoid species [16], [17]. The subclass division has found additional support by several independent characters, such as different larvae types and distinctive development in both subclasses (coeloblastula in Calcinea, amphiblastula with special development in Calcaronea, see [17]). Furthermore, the two subclasses can be distinguished by different ratios of isotopes incorporated into the spicules during bio-mineralization [18] and by the analyses of small subunit (SSU) and partial large subunit (LSU) ribosomal RNA genes (rDNA) [6], [19], [20].

### Calcinea

Hartman corroborated the Calcinea-Calcaronea concept and provided an order-level taxonomy [17]. In Calcinea, he proposed three orders: (1) Clathrinida for homocoel Calcinea without a cortex, (2) Leucettida for heterocoel Calcinea with cortex or dermal membrane and (3) Pharetronida Zittel 1878 for leuconoid Calcarea with a reinforced skeleton of fused spicules or formed by an aspicular network. However, Vacelet [21] showed that some members of Pharetronida belong to Calcaronea, whereas others belong to Calcinea, for which he proposed the orders Lithonida and Murrayonida, respectively [22]. In the latest revision of Calcinea [23], Leucettida was rejected, because the transition from simple homocoel Calcinea to heterocoel Calcinea was interpreted to have occurred independently several times and in different evolutionary pathways [13], [23]. All Calcinea with free spicules were placed in the order Clathrinida [23]. It has to be noted, that none of the proposed independent evolutionary lines in Calcinea were based on phylogenetic analyses, and as such they are debatable despite being presented in a logical and convincing way.

### Calcaronea

Within Calcaronea, Hartman [17] placed homocoel sponges without cortex and dermal membrane in his order Leucosolenida and heterocoel calcaroneans in the order Sycettida Bidder 1898. The current order level taxonomy [24] differs not only by containing the order Lithonida but also by proposing a new order, Baerida, for Calcaronea with skeletons formed exclusively or in substantial parts by microdiactines [24]. Sycettida was rejected, and its species (with the exception of Baerida) were included in Leucosolenida [24].

### Phylogenies based on morphological and DNA data

The first comprehensive phylogenetic analysis of morphological characters was performed by Manuel, et al. [19], who showed that little phylogenetic information is present and suggested that morphological characters contain a high level of homoplasy. The analyses of ribosomal rRNA genes found strong support for the monophyly of the two subclasses Calcinea and Calcaronea, but also revealed that many of the classically recognized taxa at the order, family and genus levels were not monophyletic, suggesting that these taxa are artificial groupings [6], [19], [20]. Unfortunately, the morphological evolution of the aquiferous system and the skeletal arrangements is difficult to understand considering the phylogenetic hypotheses obtained from molecular phylogenies.

To clarify the evolution of this group of sponges, we included several additional critical taxa in our analyses, especially from members of the families Leucaltidae (Calcinea), Grantiidae and Heteropiidae (both Calcaronea) and analysed a concatenated dataset of the complete SSU rDNA and nearly the complete LSU rDNA. For the latter, previously available nucleotide positions of partial LSU rDNA [20] were substantially increased. We analyzed our data under various models of RNA specific substitution models and used the resulting phylogenies to evaluate different hypotheses.

## Materials and Methods

### Sample collection and species identification

Calcareous sponge specimens were collected in the Red Sea (Gulf of Aqaba), with kind permission from the Egyptian Environmental Affairs Agency (EEAA), and in the Great Barrier Reef, with kind permission from the Great Barrier Reef Marine Park Authority (Permit nos G98/142, G98/022, G00/638, G06/16547.1). Additional specimens were obtained from museum collections (Tables 1, 2). To determine the sponges we examined the skeletal arrangements and the nature of the aquiferous system in thin sections, which were prepared as follows.

**Table 1:** Included specimens of Calcinea, their sample localities and GenBank accession numbers.

Species	Family	Voucher	Locality	SSU	LSU
**Clathrinida**					
*Clathrina adusta* *	Clathrinidae	QM G313665	GBR, Wisatri Reef	AM180962	**JQ272288**
*Clathrina cerebrum*	Clathrinidae	–	–	U42452	AY563541
*Clathrina helveola* *	Clathrinidae	QM G313680	GBR, Heron Reef	AM180958	**JQ272291**
*Clathrina luteoculcitella* *	Clathrinidae	QM G313684	GBR, Channel Wistari/Heron Reef	AM180959	**JQ272283**
*Clathrina* sp.	Clathrinidae	QM G313693	GBR, Yonge Reef	AM180960	**JQ272286**
***Calthrina*** ** sp. GW957**	Clathrinidae	GW 975	GBR, Mac's Reef	**JQ272310**	**JQ272285**
*Clathrina* wistariensis	Clathrinidae	QM G313663	GBR, Wistari Reef	AM180961	**JQ272303**
*Guancha* sp.	Clathrinidae	QM G316033	GBR, Rene's Nook	AM180963	**JQ272284**
*Soleneiscus radovani* *	Soleneiscidae	QM G313661	GBR, Wistari Reef	AF452017	**JQ272289**
*Soleneiscus stolonifer*	Soleneiscidae	QM G313668	GBR, Wistari Reef	AM180955	**JQ272290**
*Levinella prolifera*	Levinellidae	QM G313818	GBR, Hook Reef	AM180956	**JQ272292**
***Ascandra*** ** sp.**	Leucaltidae	QM G323326	Tasmania, King Island Canyons	n.a.	**JQ272293**
*Leucaltis clathria* #	Leucaltidae	QM G316022#	GBR, DJ's reef	AF452016	**JQ272302**
***Leucettusa*** ** sp. 1**	Leucaltidae	QM G323232	Tasmania, Ling Hole	**JQ272311**	**JQ272300**
***Leucettusa*** ** sp. 2**	Leucaltidae	QM G323283	Tasmania, Ling Hole	n.a.	**JQ272299**
***Leucettusa*** ** sp. 2**	Leucaltidae	QM G323253	Tasmania, King Island Canyons	n.a.	**JQ272301**
*Ascaltis* sp.	Leucascidae	QM G313824	South Pacific, Pitcairn Islands	AM180957	**JQ272287**
*Leucascus* sp.	Leucascidae	QM G316051	GBR, Hook Reef	AM180954	**JQ272305**
*Leucetta chagosensis*	Leucettidae	QM G316279#	Coral Sea, Osprey Reef	AF182190	**JQ272296**
*Leucetta microraphis*	Leucettidae	QM G313659	GBR, Wistari Reef	AM180965	**JQ272297**
*Leucetta* sp.	Leucettidae	QM G313691	GBR, Yonge Reef	AM180964	**JQ272298**
*Leucetta villosa* *	Leucettidae	QM G313662	GBR, Wistari Reef	AM180966	**JQ272295**
*Pericharax heteroraphis*	Leucettidae	QM G316295	Coral Sea, Holmes Reef	AM180967	**JQ272294**
**Murrayonida**					
*Murrayona phanolepis*	Murrayonidae	QM G313992	Coral Sea, Osprey Reef	AM180968	**JQ272304**
*Lelapiella incrustans*	Lelapiellidae	QM G313914	Vanuatu	AM180969	**JQ272306**

New specimens and sequences are in bold.

* = Specimen is the holotype;

# = SSU sequence comes from another individual (GenBank).

**Table 2:** Included specimens of Calcaronea, their sample localities and GenBank accession numbers.

Species	Family	Voucher	Locality	SSU	LSU
**Baerida**					
*Petrobiona massiliana* #	Petrobionidae	–	Mediterranean, Marseille	AF452026	**JQ272307, JQ272308**
*Eilhardia schulzei*	Baeridae	QM G316071	GBR, Mac's reef	AM180980	**JQ272256**
*Leuconia nivea*	Baeridae	–	–	AF182191	AY563534
**Lithonida**					
*Plectroninia neocaledoniense*	Minchinellidae	QM G316300	Coral Sea, Holmes Reef	AM180979	**JQ272309**
**Leucosolenida**		–	–		
*Leucosolenia* sp.	Leucosolenidae	–	–	AF100945	AY026372
*Sycon capricorn*	Sycettidae	QM G316187	GBR, Ribbon Reef 3	AM180970	**JQ272272**
***Sycon carteri***	Sycettidae	SAM PS 0142	Australia, Ulladulla	**JQ272314**	**JQ272260**
*Sycon ciliatum*	Sycettidae	–	–	AJ627187	AY563532
*Sycon raphanus*	Sycettidae	–	–	AF452024	AY563537
*Grantia compressa*	Grantiidae	–	–	AF452021	AY563538
***Teichonopsis labyrinthica***	Grantiidae	SAM PS 0228	South Australia, Kangaroo Island	**JQ272317**	**JQ272264**
*Ute amupllacea* *	Grantiidae	QM G313669	GBR, Wistari Reef	AM180972	**JQ272266**
***Ute*** ** aff. ** ***syconoides*** ** 1**	Grantiidae	QM G323233	Tasmania, King Island Canyons	**JQ272319**	**JQ272269**
***Ute*** ** aff**. ***syconoides*** ** 2**	Grantiidae	QM G313694	GBR, Yonge Reef	**JQ272318**	**JQ272271**
***Ute*** ** aff. ** ***syconoides*** ** 3**	Grantiidae	GW 975	GBR, Lizard Island	**JQ272320**	**JQ272270**
***Synute pulchella***	Grantiidae	WAM Z1404	West Australia, Reru Island	**JQ272316**	**JQ272274, JQ272275**
*Leucandra aspera*	Grantiidae	–	–	AF452022	AY563535
*Leucandra nicolae* *	Grantiidae	QM G313672	GBR, Wistari Reef	AM180974	**JQ272268**
*Leucandra* sp.	Grantiidae	QM G316285	Coral Sea, Osprey Reef	AM180971	**JQ272265**
***Aphroceras*** ** sp.**	Grantiidae	SAM PS 0349	Tasmania, Waterfall Bay	**JQ272315**	**JQ272273**
*Leucascandra caveolata*	Jenkinidae	QM G316057	GBR	AM180973	**JQ272259**
*Anamixilla toressi*	Jenkinidae	–	–	AF452020	AY563536
*Syconessa panicula*	Heteropiidae	–	–	AM180976	**JQ272276**
***Sycettusa*** ** aff. ** ***hastifera***	Heteropiidae	GW 893	Red Sea, Gulf of Aqaba	**JQ272322**	**JQ272282**
***Sycettusa*** ** cf. ** ***simplex***	Heteropiidae	ZMA POR11566	Western Indian Ocean, Amirantes	**JQ272321**	**JQ272279,** **JQ272280**
*Sycettusa tenuis*	Heteropiidae	QM G313685	GBR, Heron Reef	AM180975	**JQ272281**
*Sycettusa* sp.	Heteropiidae	–	–	AF452025	AY563530
*Vosmaeropsis sp.*	Heteropiidae	–	–	AF452018	AY563531
***Grantessa*** ** sp. 1**	Heteropiidae	GW 974	GBR, Lizard Island	**JQ272313**	**JQ272277**
***Grantessa*** ** sp. 2**	Heteropiidae	GW 979	GBR, Lizard Island	**JQ272312**	**JQ272278**
***Leucilla*** ** sp.**	Amphoriscidae	ZMA POR5381	Caribbean, Netherlands Antilles	**JQ272323**	**JQ272257, JQ272258**
*Paraleucilla magna* #	Amphoriscidae	GW 824	Brazil, Arailal de Cobo	AF452023	**JQ272267**
***Grantiopsis cylindrica***	Lelapiidae	GW 973	GBR, Lizard Island	**JQ272324**	**JQ272263**
*Grantiopsis heroni* *	Lelapiidae	QM G313670	GBR, Wisatri Reef	AM180975	**JQ272261**
*Grantiopsis sp.*	Lelapiidae	QM G313969	Coral Sea, Osprey Reef	AM180977	**JQ272262**

New specimens and sequences are in bold.

* = Specimen is the holotype;

# = SSU sequence comes from another individual (GenBank).

Parts of the sponges preserved in 70–96% ethanol (EtOH) were gradually transferred to 30% EtOH in water over a dilution series (70%, 50%, 30% EtOH). Tissues were then stained overnight in a 30% EtOH-fuchsine solution. The stained tissue was dehydrated in a dilution series (50%, 70%, 90%, 99% EtOH-fuchsine-solution). For embedding, the EtOH-fuchsine solution was gradually replaced with LRwhite resin (in dilution steps of 33%, 50%, 66%, 100% LRwhite, all at 4°C to prevent polymerization; the last step had an overnight incubation). For final embedding, LRwhite was exchanged, and after one hour of incubation at 45°C polymerization was induced at 60°C overnight. From the resulting block, we took sections of suitable thickness (10–500 µm; starting with a 200 µm section) from the block with a Leica 1600 saw microtome (Leica, Nußloch, Germany). To stain the cells and nuclei on the surface of the section, we suspended the section for 1∶30 min to a 30% EtOH-Touledein blue and 30% basic fuchsine solution; then, we immediately washed off the dye with water. Dried and stained sections were mounted on microscopic slides with Eukitt (Fluka). Spicules were obtained either from the lysis step from the DNA extraction (see below) or by dissolution of tissue with sodium hypochlorite. The obtained spicules were washed five times with water and transferred to a microscopic slide, dried, and mounted with Eukitt. Sections and spicule preparations were observed and documented on a Zeiss Axiolab Microscope equipped with a Canon PowerShot G2 digital camera. The identification of calcarean genera followed available keys [25]. When possible, species were identified by comparing original descriptions to our specimens. Habitus and sections of these newly included specimens are included here or in Figs. S1 and S2.

The re-examination of two specimens included in a previous study leads us to the conclusion that the two specimens were incorrectly determined at the generic level. The specimen QM G313824 was previously considered to be *Clathrina cerebrum.* We find that the specimen belongs to the genus *Ascaltis*, because it possesses a (thin) cortex and a large central cavity. Another specimen (QM G316285) was previously identified as *Aphroceras* sp. Although this specimen does possess larger diactines, they are not longitudinally arranged and do not support the cortex as in *Aphroceras*. We identified this specimen as *Leucandra* sp.

The identification of another specimen (SAM-PS0349) was also problematic. It clearly belongs to Grantiidae and possesses large longitudinal diactines that support the cortex and the atrial skeleton. This arrangement is typical of members of the genus *Amphiute* [26]. But although a syconoid aquiferous system is a diagnostic character for this genus, our specimen shows a leuconoid organization. Therefore, we decided to classify it as *Aphroceras* sp. *Aphroceras* is defined by longitudinal diactines in the cortex and a leuconoid aquiferous system [26], thus its diagnosis does not explicitly exclude the presence of diactines associated with the atrial skeleton.

### DNA extraction, PCR, sequencing and alignment

DNA was extracted with the DNeasy tissue kit (QIAGEN) or by standard phenol-chloroform extraction. Template DNA was used in dilutions of 1∶1 to 1∶500 in PCR reactions, depending on the DNA quantity and quality. Because many of the samples from museum collections yielded only highly degraded DNA, it was necessary to amplify SSU rDNA and LSU rDNA in two and up to five smaller fragments, respectively. PCRs were conducted with the BioTaq (BioLine) as previously described for SSU rRNA [20] and for LSU rRNA with different combinations of the primers, which are given in Table S1. Purified PCR products were sequenced after cycle sequencing with BigDye Terminator.3.1 (Applied Biosystems) on an ABI 3100 capillary sequencer (Applied Biosystems). Consensus sequences were created in CodonCode Aligner (http://codoncode.com) and submitted to GenBank (SSU: JQ272310–JQ272324; LSU: JQ272256–JQ272309, see Tables 1, 2.). Occasionally, it was not possible to amplify all SSU or LSU fragments for a given sample or the sequences of different fragments did not overlap. In such cases, we combined the sequences by aligning them to the most similar full LSU rRNA sequence, and recoded the missing parts as gaps.

Additional SSU rDNA and LSU rDNA sequences from Calcarea and 41 outgroup taxa were downloaded from GenBank (http://www.ncbi.nlm.nih.gov/; Tables 1, 2 for Calcarea and Table S2 for the outgroup taxa). Outgroup sequences were only included, when both SSU and LSU sequences were available in almost full length (with the exception of hexactinellid 28S sequences due to limited availability). We aligned the sequences in Seaview [27], taking into account secondary structure information (18S: [28]; 28S: [29]). The considered LSU rRNA secondary structure of a typical calcinean sequence is provided in Fig. S3. For our analyses, the C-Domain in LSU was excluded for the outgroup taxa and coded as ‘gaps’ in the alignment, because the homology of sites among all taxa could not be established with certainty. Thus, it was possible to keep calcarean sites of this highly variable region in the analyses. Further sites of uncertain homology were removed from our alignment, and custom-made PERL scripts [28] were used to generate input files that included secondary structure information suitable for PHASE [30], [31] and RAxML v. 7.2.8 [32].

### Phylogenetic analysis

Most phylogenetic methods assume that characters in a data matrix evolve independently from each other, but this assumption is clearly violated in the helices of rRNA because paired nucleotides coevolve driven by the selection pressure to maintain the secondary structure, which is pivotal for rRNA function within the ribosome [33]–[38]. By neglecting these coevolutionary processes, phylogenetic inferences can be biased and result in suboptimal tree topologies (e.g., [33], [38]–[40]). Solutions to this problem are special evolutionary models, which instead of single bases consider the two paired bases of helices, the so-called doublet, as single characters. Such models have been shown to outperform standard 4×4 models of nucleotide evolution in analyses of rDNA data [38]–[44]. Several doublet models that make different assumptions about the evolution of doublets have been proposed (for a comprehensive overview see [37]).

In contrast to standard 4×4 models of nucleotide substitution, the paired nucleotides in an RNA helix are the single characters in doublet models. Three families of doublet models can be distinguished according to the number of recognized doublets [37]. In 16-state models, all possible pairs are considered. The likelihood is calculated in a 16×16 matrix, resulting in a general reversible model with 119 free rate parameters and 15 free frequency parameters. Such a high number of parameters make general reversible 16-state models impractical to use [37]. Moreover, because mismatch base pairs (MM), i.e. pairs other than Watson-Crick pairs and GU/UG pairs, are rare in real RNA data, these states are pooled into one class (MM) in 7-sate models, or completely ignored in 6-state models. Each model family has a number of different models, which through restrictions and assumptions reduce the number of parameters compared to the most general model. A study with a 5-taxon data set compared the models within each model family and suggested that the most general models are to be preferred over restricted ones [37]. However, comparisons among the doublet model families are not possible because not only the model parameters but also the data matrices are different [37]. We applied 6-, 7- and 16 state models in a likelihood framework using RAxML 7.2.8 [32] and in Bayesian inference using the PHASE software (www.bioinf.manchester.ac.uk/resources/phase/index.html).

For our analyses, we used a concatenated dataset of SSU and LSU rDNA (4,939 positions). Previous studies with data from SSU and a smaller LSU fragment have shown that the combination of both genes lead to a finer phylogenetic resolution, compared to single gene analyses (especially with SSU DNA [6], [20]). Furthermore, SSU and LSU rRNA are parts of the ribosomal cistron, which during transcription is transcribed into one pre-rRNA before the splicing of the internal transcribed spacer regions (ITS). We partitioned the combined dataset into two partitions called stem (paired sites) and loop (unpaired sites). For an analysis with a standard 4×4 model under ML, we also applied a different partitioning scheme with one partition for SSU and one for LSU rDNA.

In PHASE, each run had a burn-in phase of 1,000,000 generations, followed by 10,000,000 sampling generations, from which every 200th tree was sampled. We used Tracer v 1.5 (http://tree.bio.ed.ac.uk/software/tracer/) to monitor the parameter sampling of each run. To transform the PHASE output files into a readable Tracer format, we modified the Perl script phase2tracer.pl from Matt Yoder (http://hymenoptera.tamu.edu/rna/download.php) to handle larger PHASE2 output files. The modified script is available on request.

In RAxML, we applied GTR models with gamma distribution to compensate for the rate heterogeneity among sites. For the stem partition, different models of each family were applied (S6A–E, S7A–E, 16A and 16B) in independent analyses using the rapid bootstrapping algorithm with 1,000 bootstrap replicates. The resulting phylogenetic trees were visualized with FigTree v.1.3.1 (http://tree.bio.ed.ac.uk/software/figtree/).

Unfortunately, no a-priori model testing software, such as jModeltest for standard models, is available yet for doublet models or partitioned datasets. Moreover, comparisons among standard models and doublet models, as well as among doublet models of different families are not possible [37]. Following the suggestion by Savill, et al. [37], we choose the 7A model to discuss most of our results and to test phylogenetic hypotheses. By using this model, we did not ignore the class of mismatches as in 6-state models, nor did we assign an own character class to each of the rare mismatch doublets as in 16-state models. To illustrate model-dependent differences in the topologies, strict consensus trees for results under each family of doublet models were calculated in PAUP* 4.0b10 [45] and are presented in Figs. S4 and S5.

We used MacClade v. 4.07 [46] to trace the evolution of morphological characters according to our phylogenetic hypothesis from the Bayesian analysis with the 7A model.

### Testing phylogenetic hypotheses

To test whether the tree topologies obtained with our data were significantly better than other phylogenetic hypotheses, we re-analyzed the dataset with RAxML and the 7A-model of nucleotide evolution under specific topology constraints of the tested taxa.

In Calcaronea, we constrained the following monophyletic taxa: (a) a clade containing Lithonida, monophyletic Baerida and monophyletic Leucosolenida (following [24]); (b) Leucosolenida Hartman 1958 and Sycettida Hartman 1958 with the modification that members of Lithonida were considered as Sycettida following Hartman's definition of the order [17]; (c) the families Amphoriscidae, Grantiidae, Heteropiidae and Jenkinidae being all monophyletic; (d–g) constraining each of these families as monophyletic: (d) Amphoriscidae; (e) Grantiidae; (f) Heteropiidae; (g) Jenkinidae.

In Calcinea, we constrained the following taxa as monophyletic: (a) monophyletic orders Clathrinida and Murrayonida [24]; (b) Murrayonida, Leucaltidae, (Clathrinidae+Leucascidae+Leucettidae), a scenario presented in [24]; (c) the order Clathrinida *sensu* Hartman [17]; and the families (d) Leucaltidae, (e) Leucascidae and (f) Clathrinidae.

ML trees under each topological constraint were obtained using RAxML (model S7A for stem regions) and the previously described settings. The resulting trees were calculated and combined with the unconstrained ML tree (S7A-model) in one file for each subclass (Calcaronea and Calcinea). RAxML was used to calculate site-specific likelihood values for these two sets of trees. Using these files, an approximately unbiased (AU) test [47] was performed in Consel [48] following the program's manual.

## Results

### Topologies from ML and BI under different models

**Figure 3:**
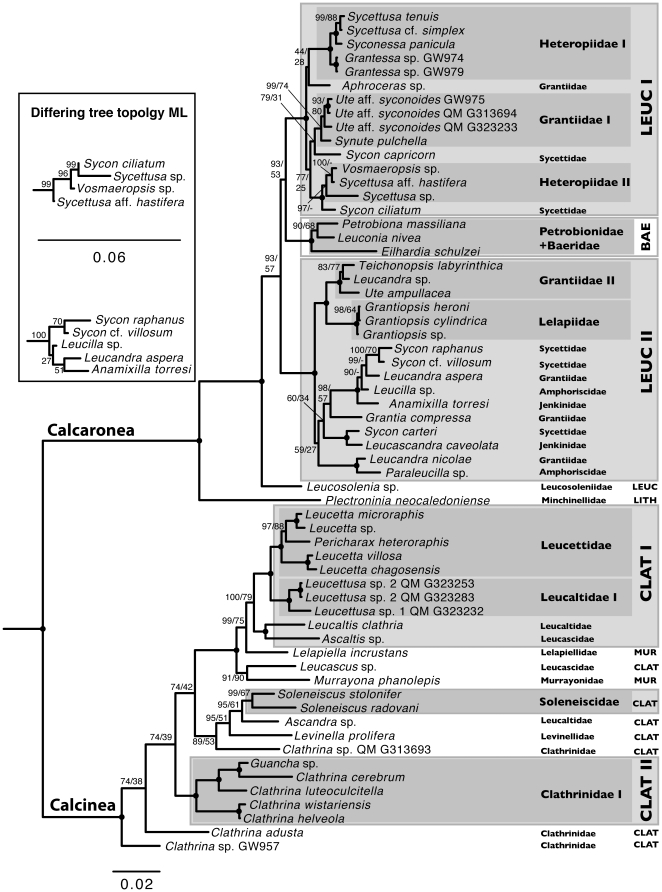
Bayesian phylogeny of Calcarea inferred with the RNA7A model for partition stem. Outgroup taxa not shown (compare Figs. S4, S5). Support values are given at the nodes (PP/BS of ML analyses under the same model). Clades are shaded and numbered for taxa that are not monophyletic. Order names are abbreviated: BAE = Baerida; CLAT = Clathrinida; LEUC = Leucosolenida; LITH = Lithonida; MUR = Murrayonida. Insert: ML topologies of two clades that differ from Bayesian inference (with BS values).

Following suggestions made by Savill, et al. [37] we present the tree topologies obtained with model 7A for stem regions in Fig. 3. In Calcarea, some minor differences between this model and other 7-state, 6-state and 16-state models mostly occurred in nodes without strong support in the presented topology (see Figs. S4, S5). Topologies obtained with ML under model 7A slightly differed from the Bayesian inference (insert, Fig. 3). Differences in the posterior probabilities (PP) and bootstrap support (BS) can be appreciated in Fig. 3. When standard GTR models were applied to the dataset partitioned in stem and loop, we observed a different position for *Leucosolenia* (occurring basal to clade LEUC I, see below), which, in the case of the Bayesian analysis (but not under ML), results in a phylogeny with a weakly supported (PP: 55) sister group relationship of Baerida and Leucosolenida (Fig. S6). When using a partitioning scheme by gene (SSU and LSU) with GTR models, the position of *Leucosolenia* is recovered as by the analyses with doublet models (Fig. S6).

### Monophyly of Calcarea and relationship to outgroup taxa

All analyses resulted in a strongly supported monophyletic Calcarea, with a subdivision into Calcinea and Calcaronea (also with high support values, Fig. 3 and Figs. S4, S5, S6). The position of Calcarea with respect to the outgroup taxa differed with the applied models and between ML and Bayesian inference; however, Calcarea was never found in a monophyletic clade Porifera. Instead, monophyletic Demospongiae and Hexactinellida were sister taxa ( = Silicea) with the homoscleomorph sponge *Oscarella* as sister taxon (with high support values in most cases). Several relationships of other outgroup taxa were strongly supported by PP and BS values and were found in all the analyses regardless of the applied model (e.g., the monophyly of the phyla Placozoa, Cnidaria and Ctenophora). Cnidaria and Placozoa were sister taxa. Otherwise the relationships among these phyla and their relationships to the sponge clades are not strongly supported and varied in the different analyses.

### Relationships of Calcarea

In most cases, our phylogeny is compatible with the results of previous rDNA analyses [19], [20]. Likewise, we found strong support for the two monophyletic subclasses Calcinea and Calcaronea. Below the subclass level, we confirm the non-monophyly of several taxa that had been previously reported as such [20]: (i) in Calcaronea, the order Leucosolenida, the families Heteropiidae, Grantiidae, Jenkinidae Sycettidae, and the genera *Sycon, Sycettusa*, *Leucandra*; (ii) in Calcinea, the orders Clathrinida and Murrayonida, the families Clathrinidae, Leucaltidae, Leucascidae and the genus *Clathrina.*

Our topology could resolve some relationships that were only recovered as polytomies by Dohrmann, et al. [20] e.g., within Leucosolenida (Calcaronea) and Leucettidae (Calcinea). In addition, several clades found in the former study were not recovered in the analyses of our extended taxon and character set. For instance, our topology does not contain Clade H1 and clade H2 in Calcaronea nor Clade K in Calcinea (Fig. 3 in [20]). With the extended taxon set, new species could be placed into the phylogeny, and we uncovered additional contradictions to the classification of some taxa.

### Relationships within Calcaronea

In Calcaronea, the only sampled species of Lithonida, *Plectroninia neocaledoniense*, is the sister taxon to a clade that comprises the other sampled Calcaronea, in which *Leucosolenia* is basally diverging, as previously reported [20]. The order Baerida (clade BAER, Fig. 3) is nested within Leucosolenida, with a sister clade relationship (PP: 93, BS: 53) to a clade of several Leucosolenida (LEUC I, Fig. 3).

The clade LEUC I comprises all sampled members of Heteropiidae (occurring in two clades Heteropiidae I and II, Fig. 3), several Grantiidae (*Ute* aff. *syconoides*, *Synute* and *Aphroceras*, which are also not recovered as a clade), and two *Sycon* species, *Sycon ciliatum* and *Sycon capricorn*. The clade Heteropiidae I contains *Sycettusa tenuis* and *Sycettusa* cf. *simplex*, which form a sister group to *Syconessa panicula*. Two *Grantessa* specimens (most likely conspecific) are the sister group to all of the latter species. *Aphroceras* sp. (family Grantiidae) is the sister group to Heteropiidae I with very low PP support.

In Heteropiidae II, *Sycettusa* aff. *hastifera* is more closely related to V*osmaeropsis* than to *Sycettusa* sp. *Sycon ciliatum* (Sycettidae) is the sister taxon to Heteropiidae II. This topology has high PP support (97–100) but is not recovered in the same way in the 7A ML analysis. In this latter analysis, the species of Heteropiidae II and *Sycon ciliatum* also form a highly supported clade, but here relationships among the species are recovered differently, with *Sycon ciliatum* nested inside a clade of Heteropiidae (Fig. 3, insert). The topology recovered with ML also finds high BS support (96–99). The relationships of the species of Heteropiidae II and *Sycon ciliatum* are therefore dependent on the method employed. However, both hypotheses are consistent with a close relationship between these heteropiid species and *Sycon ciliatum* and agree that *Sycettusa* sp. and *Sycettusa* cf. *simplex* do not form a monophyletic group. Because two other included species of *Sycettusa* are also present in the clade Heteropiidae I, the non-monophyly of the genus is out of the question, regardless of the relationships within Heteropiidae II.

**Figure 4:**
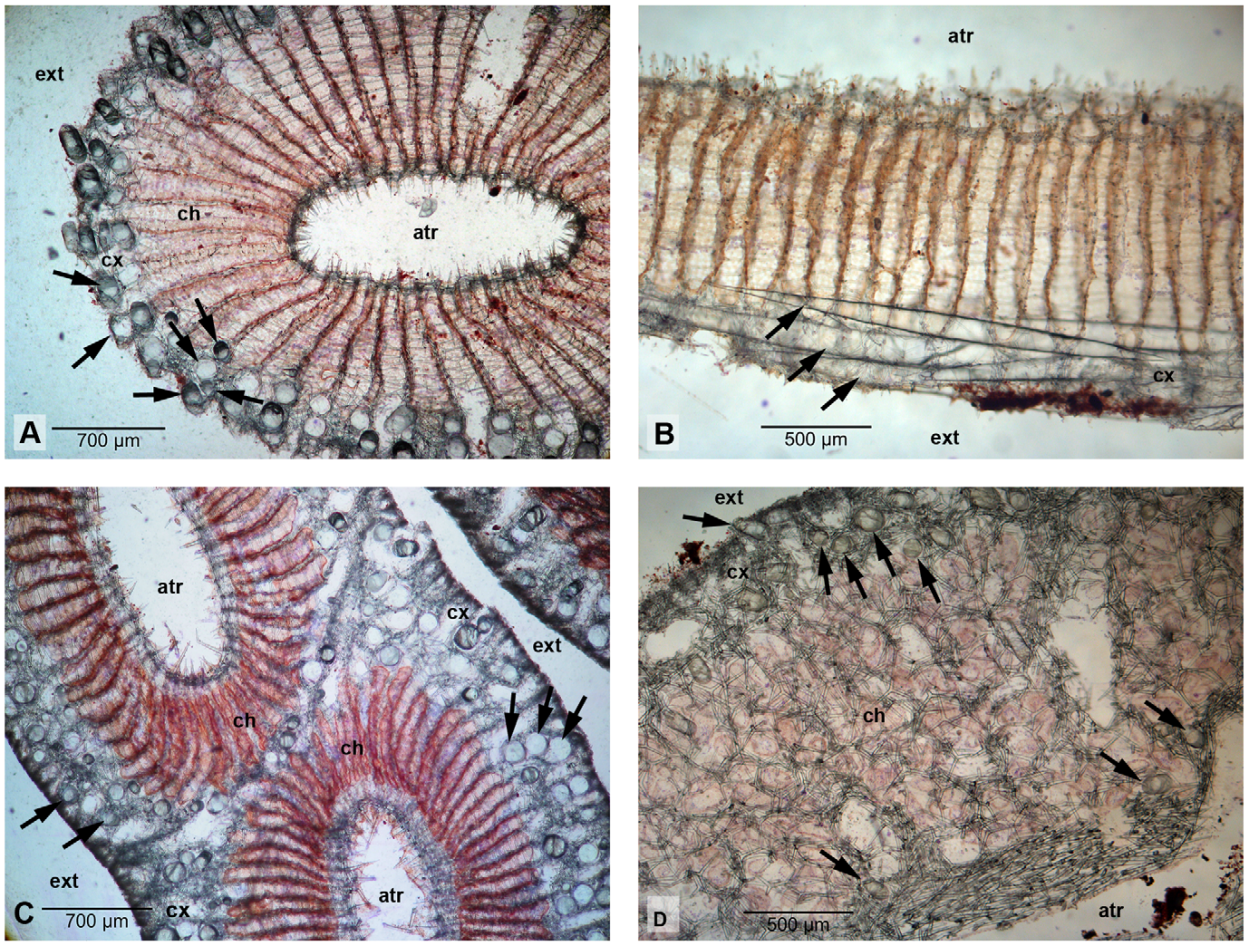
Skeletal organization of Grantiidae of clade LEUC I. A,B: *Ute* aff. *syconoides* (GW975) in cross section (A) and longitudinal section (B); C: Cross section of *Synute pulchella*; D: Cross section of *Aphroceras* sp. Arrows point to the giant longitudinal diactines. atr = atrium; ch = choanosome; cx = cortex, ext = exterior of the sponge.

The clade Grantiidae I contains a clade of three specimens of *Ute* aff. *syconoides* with *Synute pulchella* as sister species, but it does not include *Aphroceras* sp. Interestingly, all of these grantiid genera in clade LEUC I have giant longitudinal diactines in their cortex (Fig. 4). However, one additional species with this feature, *Ute ampullacea*, is found in LEUC II and is not closely related to *Ute* aff. *syconoides*. Grantiidae I and *Sycon capricorn* (Sycettidae) form a clade (with high PP but low BS support), which itself is sister group to (Heteropiidae I+*Sycon ciliatum*), but with low support (PP: 77; BS: 25).

In Baerida, *Eilhardia schulzei* is the sister taxon to (*Petrobiona massiliana*+*Leuconia nivea*), which results in Baeridae as a non-monophyletic clade.

In LEUC II, Grantiidae II and Lelapiidae form a clade with high support. Grantiidae II comprises *Teichonopsis*, *Ute ampullacea* and *Leucandra* sp. Considering that *Ute* aff. *syconoides* falls in clade LEUC I, the genus *Ute* is clearly not monophyletic. The genus *Leucandra* is also paraphyletic because *Leucandra nicolae* and *Leucandra aspera* are neither in a close relationship to each other nor to *Leucandra* sp. Within the remaining taxa of clade LEUC II, Jenkinidae (*Anamixilla* and *Leucascandra*), Amphoriscidae (*Leucilla* and *Paraleucilla*) and additional taxa of Sycettidae (genus *Sycon*) and Grantiidae (*Leucandra, Grantia*) are clearly all non-monophyletic. While *Sycon raphanus* and *Sycon* cf. *villosum* are sister taxa, *Sycon carteri* is most closely related to *Leucascandra caveolata* form the family Jenkinidae. However, *Anamixilla toressi*, the only other included species of Jenkinidae in the dataset, is more closely related to *Leucilla* (Amphoriscidae), *Leucandra aspera* (Grantiidae) and the previously mentioned *Sycon raphanus* and *Sycon* cf. *villosum*. *Grantia compressa* is the sister taxon to the clade including the latter species (PP 98, BS: 57). *Leucandra nicolae* and *Paraleucilla* sp. form a highly supported clade, but the position of this clade, as shown in Fig. 3, finds only low support from the data (PP: 59, BS: 27).

With the presented relationships, many classically recognized taxa of Calcaronea are not monophyletic: the order Leucosolenida; the families Heteropiidae, Grantiidae, Jenkinidae Sycettidae, Amphoriscidae and Baeridae; and the genera *Sycon, Sycettusa*, *Leucandra* and *Ute*.

#### Testing phylogenetic hypotheses in Calcaronea

Our phylogenetic test (Table 3) shows that the classification of Calcaronea into the three monophyletic orders Lithonida, Baerida and Leucosolenida was not supported by our data. The same occurred with the tested families Amphoriscidae, Grantiidae, Heteropiidae and Jenkinidae. The only hypothesis that could not be rejected was the taxonomic scheme of Hartman 1958, which separates homocoel and heterocoel Calcaronea into his orders Leucosolenida and Sycettida.

**Table 3:** p-values for the approximately unbiased test [47] for different topological constrains in Calcaronea.

	Constrained monophyly	ln ML	*P* AU	reject hypotheses
	Unconstrained (ML)	−46409.003048	**0.952**	no
a	Lithonida, Baerida, Leucosolenida	−46582.726312	**3e-09**	yes
b	Leucosolenida sensu Hartmann 1958, Sycettida sensu Hartman 1958	−46443.927802	**0.087**	no
c	Amphoriscidae, Heteropiidae, Jenkinidae, Grantiidae	−47086.355629	**5e-10**	yes
d	Amphoriscidae	−46564.717608	**2e-05**	yes
e	Grantiidae	−46895.066357	**0.002**	yes
f	Heteropiidae	−46452.767514	**0.047**	yes
g	Jenkinidae	−46539.002443	**0.001**	yes

The hypothesis (constrained monophyly) can be rejected for p-values <0.05.

### Relationships within Calcinea

#### Calcinean orders

In the subclass Calcinea, the order Murrayonida, represented by *Murrayona phanolepis* and *Lelapiella incrustans*, is not monophyletic and both species are nested in Clathrinida (*Murrayona phanolepis* forms a low supported clade with *Leucascus* sp., and *Lelapiella* is the sister group to clade CLAT I, see below).

At the base of Calcinea, the relationships presented in Fig. 3 did not find high support (PP of 74 and BS <50). As such, the position of the root within Calcinea remains uncertain. However, Bayesian and ML trees obtained with the 7A model resulted in the same topology. Accordingly, two *Clathrina* species branched off subsequently (*Clathrina* sp. GW975 and *Clathrina adusta*), followed by clade Clathrinidae I (or CLAT II, Fig. 3), which comprises four additional *Clathrina* species (*C. helveola*, *C. wistariensis*, *C. luteoculcitella*, *C. cerebrum*) and *Guancha* sp. Next, a clade containing an additional *Clathrina* species and members of *Ascandra*, *Levinella* and *Soleneiscus* branched off. All of these taxa share an asconoid aquiferous system, i.e., they are homocoel, and lack a cortex.

More resolution is present in the remaining Calcinea, which are all characterized by the possession of a cortex and, with the exception of *Ascaltis* sp., they are heterocoel with syconoid, leuconoid or solenoid aquiferous systems. These species form a strongly supported clade, including the mentioned members of Murrayonida. In this clade, Leucettidae is monophyletic, while the genus *Leucetta* is not. *Leucetta microraphis* and *Leucetta* sp. are more closely related to *Pericharax heteroraphis* than to the clade of *Leucetta chagosensis* and *Leucetta villosa*. This relationship finds high PP and BS support.

Compared to previous studies, we included additional taxa from two genera of the family Leucaltidae (Order Clathrinida): *Ascandra* sp. and three specimens representing two undetermined species of the genus *Leucettusa*. None of the genera are closely related to each other or to the other included species of Leucaltidae, *Leucaltis clathria*; therefore, the family is polyphyletic. *Ascandra* is associated with *S*o*leneiscus* (Soleneiscidae) and *Levinella* (Levinellidae); thus, it is closely related to other taxa with an asconoid grade of organization. In contrast, the *Leucettusa* species form a monophyletic sister group to Leucettidae and, together with this latter family, a sister clade to another clade formed by *Leucaltis clathria* with *Ascaltis* sp. (Leucascidae). The position of *Ascaltis* and *Leucascus* (with *Murrayona*, see above) suggests that Leucascidae is not monophyletic.

Our phylogeny shows several non-monophyletic taxa: the orders Clathrinida and Murrayonida, the families Clathrinidae, Leucaltidae, Leucascidae and the genera *Clathrina* and *Leucetta*.

#### Testing phylogenetic hypotheses in Calcinea

The results of the AU test are presented in Table 4. The separation of Calcinea into the orders Clathrinida and Murrayonida was rejected according our data. Moreover, a subdivision into three monophyletic lineages (i) Murrayonida, (ii) Leucaltidae and (iii) (Clathrinidae+Leucascidae+Leucettidae), needed to be disregarded. These lineages were supposed to have independently gained a more complex aquiferous system from asconoid ancestors according to Borojevic, et al. [23]. Likewise, the monophyly of Leucaltidae alone had to be rejected. The contrasting scheme of Hartman [17], which classified homocoel Calcinea into one order (Clathrinida *sensu* Hartman), was not recovered in our ML and Bayesian analyses but could not be excluded as a possible scenario from our dataset. Similarly, a tree topology with monophyletic Leucascidae or monophyletic Clathrinidae cannot be omitted according to our AU test.

**Table 4:** p-values for the approximately unbiased test [47] for different topological constrains in Calcinea.

	Constrained monophyly	ln ML	*P* AU	reject hypotheses
	Unconstrained (ML)	−46409.003048	**0.865**	no
a	Clathrinida, Murrayonida	−46464.045648	**0.008**	yes
b	Murrayonida, Leucaltidae, (Clathrinidae+Leucascidae+Leucettidae)	−46773.382665	**2e-71**	yes
c	Clathrinida sensu Hartman 1958	−46420.511855	**0.361**	no
d	Leucaltidae	−46588.330103	**1e-46**	yes
e	Leucascidae	−46448.500818	**0.067**	no
f	Clathrinidae	−46437.161025	**0.091**	no

The hypothesis (constrained monophyly) can be rejected for p-values <0.05.

#### Evolution of morphological characters

**Figure 5:**
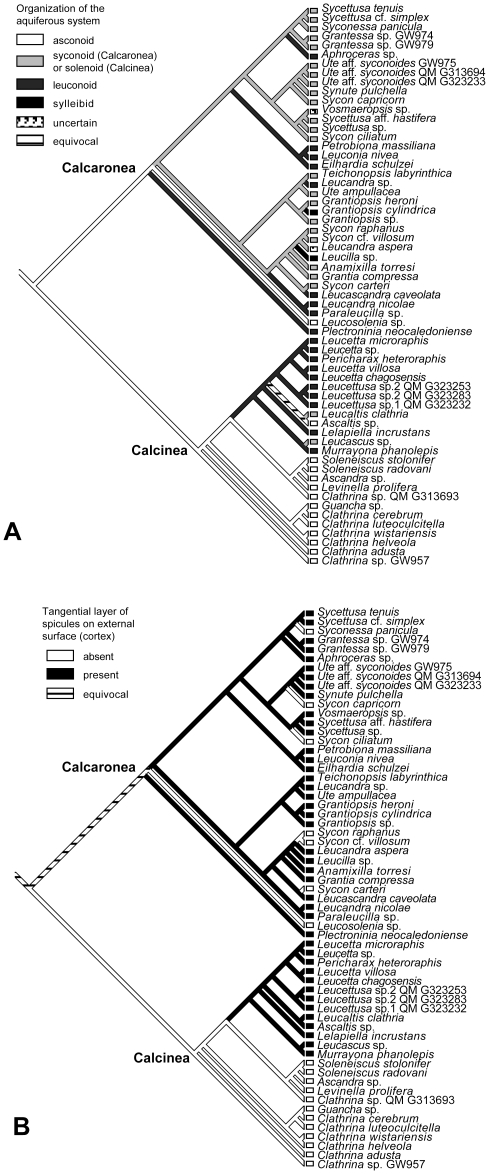
Evolution of morphological characters. A: Organization of the aquiferous system; B: Evolution of a cortex. Tree topology identical to Fig. 3.

A parsimony-based character mapping on the phylogenetic tree suggests a complex evolution of certain morphological characters (Fig. 5, Fig. S7).

In Calcaronea, leuconoid aquiferous systems have evolved several times independently from ancestral syconoid stages. The cortex was lost several times in the polyphyletic *Sycon* species and *Syconessa*. A subcortical or cortical layer of pseudosagittal spicules, a diagnostic character for the family Heteropiidae, was reconstructed to have evolved two times independently from articulated ancestors (Fig. S7). However, after collapsing nodes with less than 90% PP support, it is also possible that it evolved once and in this case was lost in *Sycon ciliatum* (Fig. S7, A). Inarticulated choanoskeletons evolved several times independently from ancestors with articulated choanoskeletons in this subclass (Fig. S7, B).

In Calcinea, the basally diverging clades are asconoid and lack a cortex. Interestingly, the acquisition of the leuconoid aquiferous systems and a cortex occurred only once at the same node according to our reconstruction (Fig. 5). The asconoid aquiferous system of *Ascaltis* has to be interpreted as a secondary reorganization of the aquiferous system. Furthermore, the solenoid *Leucascus* seems to descend from leuconoid ancestors.

## Discussion

### Polyphyly of Leucaltidae and Murrayonida and implications for morphological evolution in Calcinea

Our obtained phylogeny and our phylogenetic tests contradict the classification and scenarios of the evolution of morphological characters in Calcinea, which have been suggested before by Borojevic and coworkers [23]. In our phylogenetic tree, the polyphyly of Leucaltidae has broad implications for the classification of Calcinea. Borojevic, et al. [23] rejected Hartman's subclass-level subdivision into homocoel, cortex-lacking Calcinea (his Clathrinida) and Leucettida (heterocoel Calcinea with a cortex or dermal membrane) and suggested that a cortex and the heterocoel organization of the aquiferous system evolved independently in different lineages. One of these lineages was Leucaltidae, in which, according to these scenarios, a more complex aquiferous system evolved by infolding of the choanoderm (as observed in *Ascandra*) and a cortex developed by formation of a secondary atrial skeleton (as present in *Leucaltis* and *Leucettusa*) [23]. Accordingly, a different lineage of Calcinea evolved a cortex and a complex aquiferous system from homocoel ancestors with a clathrinoid organization (cormus of branching and anastomosed tubes as the ones observed in *Clathrina* and *Guancha*), through the formation of a cortex (organization as in *Ascaltis*) to heterocoel sponges with solenoid to leuconoid aquiferous systems (i.e., Leucascidae and Leucettidae) [23]. A third independent gain of the cortex and heterocoel organization was suggested in the order Murrayonida [23].

Our data exclude the monophyly of each of these groups. We found homocoel species branching off first in Calcinea. In addition, *Ascandra* was closely related to the other homocoel and cortex-lacking genera, *Clathrina*, *Levinella* and *Soleneiscus*, rather than to the heterocoel species of *Leucettusa* and *Leucascus*. Our phylogenetic tree contains a highly supported clade of cortex-bearing Calcinea, which are also heterocoel with the exception to *Ascaltis*. Here, *Leucascus* shows affinities to *Murrayona*, while *Leucettusa* is the sister group to Leucettidae. The tracing of character evolution suggests that a cortex and a heterocoel water system were gained once in this subclass in an ancestor of the extant cortex-bearing Calcinea and that the asconoid water system of *Ascaltis* is the result of a secondary modification (Fig. 5). This clade of Calcinea with a cortex includes *Murrayona phanolepis* and *Lelapiella* sp. (non-monophyletic Murrayonida), but otherwise it is largely congruent with Hartman's Leucettida [17]. Only the inclusion of *Ascaltis* would require a modification to his definition of this order. Accordingly, we found that Leucettida *sensu lato* could be defined as follows:

Order Leucettida Hartmann 1958 emended.

#### Diagnosis

Calcinea with a cortex.

#### Remarks

Species previously placed in Murrayonida are included in Leucettida. Leucettida contains almost exclusively heterocoel Calcinea, with *Ascaltis* being the only known exception. The asconoid aquiferous system of *Ascaltis* is interpreted as resulting from a secondary modification rather than being a primitive state.

Unfortunately, the relationships among homocoel Calcinea are not highly supported despite the extended character set compared to previous analyses. Clathrinida *sensu* Hartman is not monophyletic in our phylogeny. However, it cannot be rejected from our data according to the AU-tests. This uncertainty hampers a comprehensive revision of the order-level classification in Calcinea.

Recently, a phylogenetic study suggested that several morphological characters, such as color and presence/absence of tetractines or spines on actines, carry phylogenetic signals in Clathrinida [49]. Certainly, several of the mentioned characters can be interpreted as diagnostic synapomorphies in the phylogeny presented by the authors. However, the study largely focused on *Clathrina* species and did not include Murrayonida, which our and a previous study [20] found to be nested within the Clathrinida. In addition to this restricted taxon sampling, the position of the root in Calcinea in this study was not highly supported, similar to the results obtained with our data. A different rooting could result in a different interpretation of the evolution of these morphological characters. It will require a larger dataset (character and taxon sampling) to test the new and valuable hypotheses of character evolution proposed by Rossi, et al. [49], but it seems that morphological characters still can provide more information than what was expected from the strong conflicts of molecular phylogenies and the current classification shown in our study. At least for certain highly supported clades, some morphological features will probably be useful to indicate close phylogenetic relationships and to serve as diagnostic synapomorphies for revised taxa.

### Order- and family-level classification in Calcaronea

Our data rejects the subdivision of Calcaronea into the three monophyletic orders Lithonida, Leucosolenida and Baerida in the currently accepted classification [24]. According to our phylogeny, Baerida is nested in the paraphyletic Leucosolenida, which is consistent with previous results [6], [19], [20]. For Lithonida, the monophyly remains to be tested by including additional taxa of this order. In contrast, an alternative order-level classification of Calcaronea suggested by Hartmann [17], with the separation of Calcaronea into Leucosolenida sensu Hartman and Sycettida cannot be completely excluded. However, Leucosolenida *sensu* Hartmann in our dataset is only represented by one *Leucosolenia* specimen. In conclusion, we cannot yet provide a satisfactory alternative classification of calcaronean orders, mainly because of the non-monophyly at the family and genus levels, which prevent the generalization of our findings for a single species to its genus or family.

Due to the non-monophyly of several families, the taxonomic value of the diagnostic characters of these families has to be doubted, e.g., the pseudosagittal spicule layer in skeletons of Heteropiidae (see below), the inarticulated choanoskeleton of Jenkinidae and the tangential tetractines supporting the cortex in Amphoriscidae.

### Close relationships of Grantiidae with giant diactines and Heteropiidae

**Figure 6:**
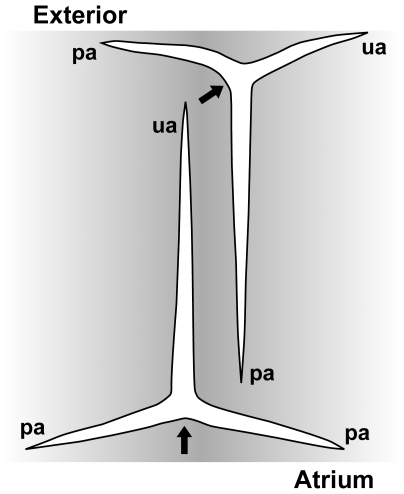
A pseudosagittal spicule and a subatrial sagittal spicule, shown in their orientation in the skeleton of Heteropiidae. The arrows point at the unpaired angles. pa: paired actine; ua: unpaired actine. Pseudosagittal spicules have an appearance similar to sagittal spicules but their paired actines are of different sizes, with one being more similar to the unpaired actine than to the other paired actine. The latter points towards the atrium.

The close relationship of Grantiidae with giant diactines in their cortex to the non-monophyletic Heteropiidae was a new finding from our data. Heteropiidae are characterized by a distal layer of a special spicule type, the pseudosagittal spicules (tri-or tetractines, Fig. 6) [24]. The polyphyly of this family implies that this character has evolved convergently at least twice or that pseudosagittal spicules were lost in other closely related species (Fig. S7, A). At first sight, pseudosagittal spicules resemble sagittal spicules (tri -or tetractines with two equal or ‘paired’ angles and one dissimilar, ‘unpaired’ angle) but differ in that one of the paired actines and the unpaired actine instead of the two paired actines are of similar length. In Heteropiidae, these spicules have a specific orientation in the skeleton, in which the equally sized unpaired and paired actines are parallel to the sponge outer surface, while the second paired actine points inwards toward the atrium (Fig. 2,A; Fig. 6). Some Heteropiidae also have longitudinal large diactines, and, in some cases, show an ‘analogous’ organization to certain genera of Grantiidae, the only difference is the possession of a layer of pseudosagittal spicules [24]. Such analogous genera of the two families are the pairs *Ute* (Grantiidae)-*Heteropia* (Heteropiidae) and *Amphiute* (Grantiidae)-*Paraheteropia* (Heteropiidae). Even in some of these Grantiidae, pseudosagittal spicules occasionally occur (e.g., in *Amphiute* [50]), but they were interpreted as the result of restricted growth of ‘normal’ spicules caused by the presence of a strong cortex [24]. In contrast to this idea, our results provide evidence that these occasional pseudosagittal spicules might indeed be homologous to the pseudosagittal spicules of Heteropiidae and that the similar skeletal organization in the mentioned pairs of genera actually reflects phylogenetic relationships. The inclusion of specimens of Heteropiidae with large diactines in molecular phylogenies would provide further insights into this question. Clearly, several genera of Heteropiidae and Grantiidae require further attention, especially the genera *Ute* and *Sycettusa*, which are polyphyletic assemblages.

### *Leucascandra* and *Sycon carteri* – implications for the evolution of inarticulated choanoskeletons

**Figure 7:**
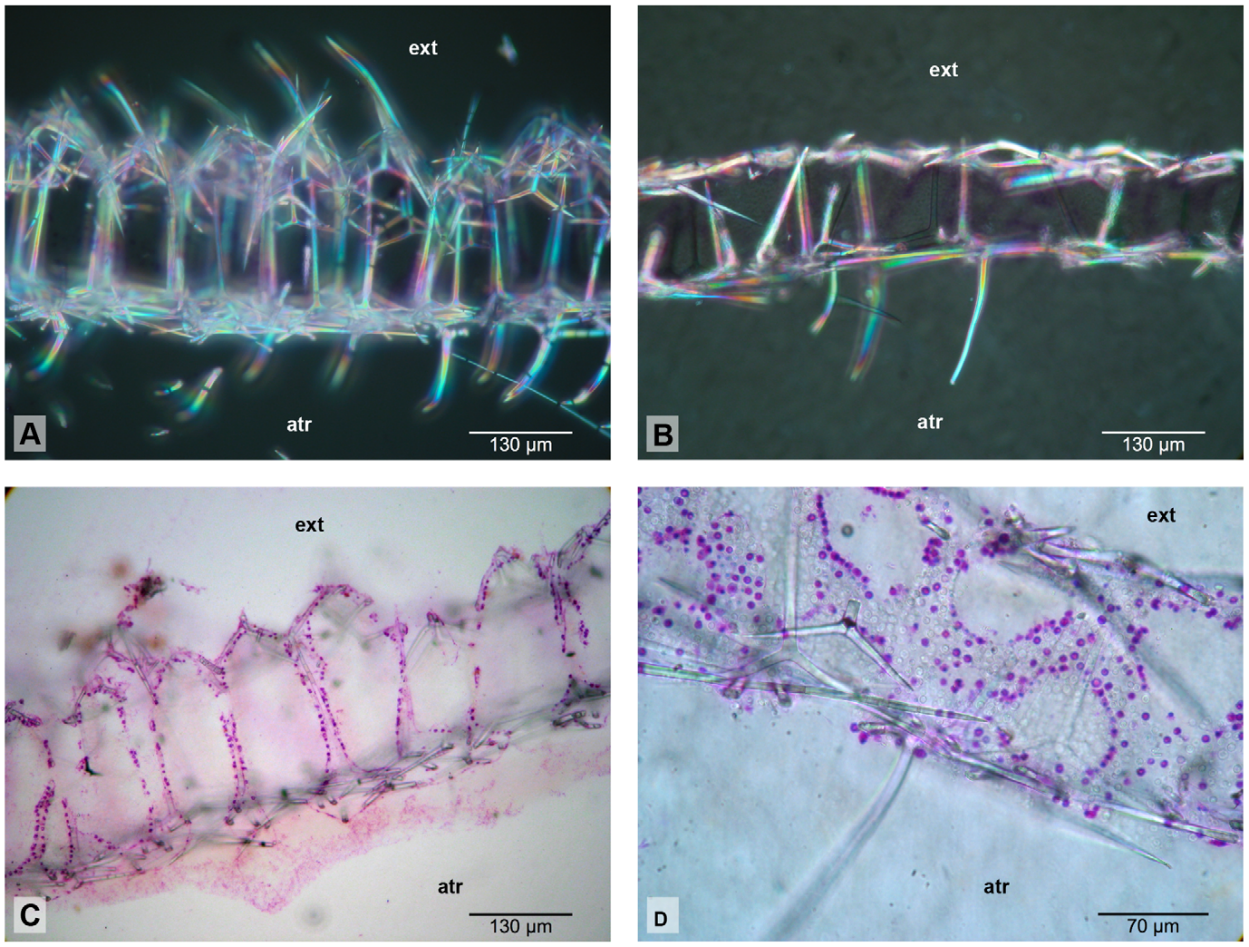
Comparison of *Sycon carteri* (A,C) and *Leucascandra caveolata* (QM G316146) (B,D). A, B: Skeletal arrangement; the atrial skeleton at the lower side, the distal cones or the cortical skeleton respectively on top, C,D: syconoid and alveolar leuconoid aquiferous system.

The sister group relationship of *Leucascandra caveoltata (Jenkinidae)* and *Sycon carteri* (Sycettidae) was unexpected because several key features appear to be obviously different in both species. For instance, the skeleton of *Leucascandra* consists of a cortex of triactines, and the inarticulated choanoskeleton contains subatrial triactines that support an irregular alveolar leuconoid choanosome (Fig. 7). In contrast, *Sycon carteri* is lacks a cortex, and its choanoskeleton forms short radial tubes with few rows of articulated triactines in the distal cones, each containing a choanocyte chamber of the syconoid aquiferous system (Fig. 7).

However, both species share characteristics, as e.g., the growth form. *Sycon carteri* is built from tubes ‘united in a copiously branching, bushy mass’ ([51], p. 79; see also Fig. S2, A), and specimens of *Leucascandra caveolata* are formed by ‘copiously branched and anastomosed tubes’ ([52], p.199). In addition, the spiculation of both species is similar (aside from the spicule size and the occurrence of diactines in the distal cones of *Sycon carteri*). In both species, the sponge wall is thin and supported almost entirely by the subatrial triactines, whose unpaired actine crosses the complete sponge wall in *Leucascandra* and reaches the distal cones in *Sycon carteri.*

The inarticulated choanoskeleton of Jenkinidae was interpreted as a primitive state rather than evolved by reduction of sponges with articulated skeletons [24]. The polyphyly of Jenkinidae (see also [20]) questions these interpretations and our data suggest that the inarticulated choanoskeleton of included species of Jenkinidae was developed twice from articulated ancestors. The close resemblance of *Sycon carteri* and *Leucascandra* sp. can be used to illustrate how ‘easily’ such transitions might be possible. One might consider a hypothetical evolution from a *Sycon-*like organization (as in *Sycon carteri*) to an inarticulated, leuconoid organization (as in *Leucascandra caveolata*) by the flattening of the distal cones so that the triactines form a cortical layer.

### Conclusions

Starting with Haeckel, the morphological diversity of different grades of complexity in extant Calcarea has repeatedly misled biologists to presume one or several evolutionary lines leading from simple to more complex forms. Our results show that the evolution of Calcarea does not follow such clear trajectories and, instead, is characterized by frequent secondary loss and convergent evolution.

The classification of Calcaronea, as understood today, is highly artificial. The fact that most orders, families and several genera are paraphyletic or polyphyletic assemblages suggests that classical revisions of such taxa (e.g., for *Clathrina,* [53]) will almost certainly exclude ‘unexpected relatives’ and, therefore, will not result in a phylogenetic classification. Yet, a basic phylogenetic framework to understand the evolution of characters in this sponge class is not available, and until it is established, any taxonomic revision should include DNA data and consider all available taxa from the given subclass. Future works should include much more species in molecular phylogenies, but not only those from missing families and genera. In addition, the use of independent molecular markers, such as mitochondrial genes, would be desirable. In Calcarea, mitochondrial sequences seem to evolve relatively fast compared to other sponges, making the genes hard to amplify with standard primers [54]; however, they would probably provide a good phylogenetic signal to resolve the nodes in our phylogeny with weak support, especially at the base of Calcinea.

At present, a revision of the higher classification is only possible for some clades because the evolution of the different organization forms is far from understood and recognizing potential diagnostic characters remains impossible. We are confident that thorough taxon sampling and DNA analysis will provide such characters in many cases, at least at shallower taxonomic levels, as indicated in a previous study focusing on *Clathrina*-species [49]. Molecular data could help to evaluate competing hypotheses and, in our case, lead to the recognition of a previously proposed order Leucettida *sensu lato*. In contrast, our study illustrates also how several relationships that were previously not conceived (such as the close relationship of *Sycon carteri* and *Leucascandra caveolata* or the sister group relationship of *Leucettusa* and Leucettidae) could be brought forward by molecular studies. Extending the available molecular and morphological dataset is crucial to finally providing a classification that is congruent with the phylogeny of this sponge group.

## Supporting Information

Figure S1 **Included new specimens of Calcinea (habitus and transversial sections). A–H:** Leucaltidae; **A, B:** *Ascandra* sp.; **C, D**: *Leucettusa* sp.1. Note the scattered small tetractines in the choanosome (D, insert); **E–H:** *Leucettusa* sp.2. E, F: QM 323253; G,H: QM 323283. Note the scattered v-shaped triactines in the choanosome (F, H, insert); H: arrow points at the apical ray of a large tetractine, which supports the choanosome; **I–J:** *Ascaltis* sp. (Leucascidae). J, insert: overview of section; K: *Clathrina* sp. (Clathrinidae) GW957. atr: atrium; chc: choanocyte chamber; cx: cortex; ext: exterior of the sponge; eh: exhalant channel; ih: inhalant channel.
(TIF)

Figure S2 **Included new specimens of Calcaronea (habitus and transversial sections).** **A:** *Sycon carteri* (in phylogeny: SAM PS0142, a conspecific specimen); **B:** *Synute pulchella* (drawn from photography); **D:** *Leucandra* sp. (transversial section); **D:** *Teichonopsis labyrinthica* (transversial section). Due to the growth-form the upper surface corresponds to the atrium. **E:** *Sycettusa* cf. *simplex* (transversial section). The arrow points at the unpaired angle of a pseudosagittal triactine. **F:** *Sycettusa* aff. *hastifera* in-situ; **G,H**: *Grantessa* sp. GW974; **I:** *Leucilla* sp. (transversial section); **J:** *Grantiopsis cylindrica.* atr: atrium; cx: cortex; ext: exterior of the sponge.
(TIF)

Figure S3 **LSU secondary structure (** ***Leucetta microraphis*** **).**
(PDF)

Figure S4 **Strict consensus of Bayesian phylogenies obtained with different doublet models of each model family.** 6-state: 6A–6F; 7-state: 7A–7F; 16-state: 16A–16F). Polytomies indicate model specific differences in tree topologies.
(TIF)

Figure S5 **Strict consensus of ML phylogenies obtained with different doublet models of each model family.** 6-state: 6A–6E; 7-state: 7A–7E; 16-state: 16A, 16B). Polytomies indicate model specific differences in tree topologies.
(TIF)

Figure S6 **Phylogenies obtained under different partitioning schemes (stem+loop, SSU+LSU) with standard, non-doublet models.** Support values (ML:BS, BI:PP) are given at the nodes. With a partitioning of stem+loop, the position of *Leucosolenia* differs from trees inferred with doublet model (Figures S4, S5), while with a partitioning into SSU+LSU, the position is as presented in Figure 3. Note also that all analyses result in a different topology at the base of Calcinea compared to our preferred doublet-inferred phylogenies (see main text).
(TIF)

Figure S7 **Evolution of pseudosagittal spicules and of the organization of the choanoskeleton.** Tree topology identical to Fig.3, only class Calcaronea is shown. **A.** Presence of a continuous layer of pseudosagittal spicules. left: obtained phylogeny, right: Excerpt of clade LEUC I with nodes of PP-support below 90% collapsed. **B.** Morphology of the choanoskeleton (characters modified from [19]). Note that inarticulated choanoskeletons evolved at least four times from ancestors with articulated choanoskeletons.
(TIF)

Table S1 **LSU rRNA primer sequences.**
(PDF)

Table S2 **GenBank accession numbers of outgroup taxa.**
(PDF)
