# Supplementary figures and images for: Molecular Phylogenetic Evaluation of Classification and Scenarios of Character Evolution in Calcareous Sponges (Porifera, Class Calcarea)
Source: PLoS One. 2012 Mar 27;7(3):e33417. doi: 10.1371/journal.pone.0033417 (PMC3314023; doi:10.1371/journal.pone.0033417)

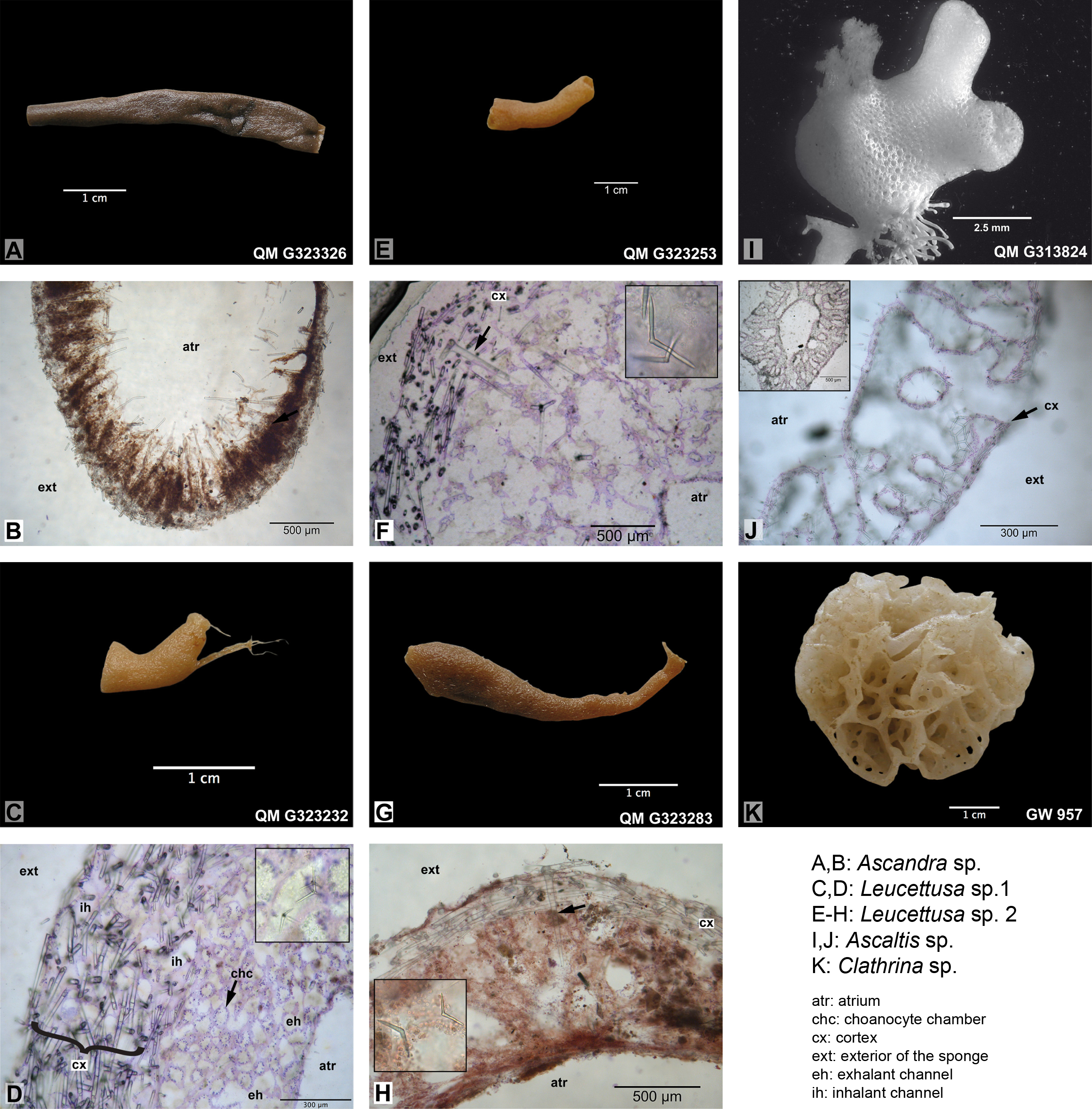

Supplement: Figure S1 — Included new specimens of Calcinea (habitus and transversial sections). A–H: Leucaltidae; A, B: Ascandra sp.; C, D: Leucettusa sp.1. Note the scattered small tetractines in the choanosome (D, insert); E–H: Leucettusa sp.2. E, F: QM 323253; G,H: QM 323283. Note the scattered v-shaped triactines in the choanosome (F, H, insert); H: arrow points at the apical ray of a large tetractine, which supports the choanosome; I–J: Ascaltis sp. (Leucascidae). J, insert: overview of section; K: Clathrina sp. (Clathrinidae) GW957. atr: atrium; chc: choanocyte chamber; cx: cortex; ext: exterior of the sponge; eh: exhalant channel; ih: inhalant channel. (TIF) [file pone.0033417.s001.tif]

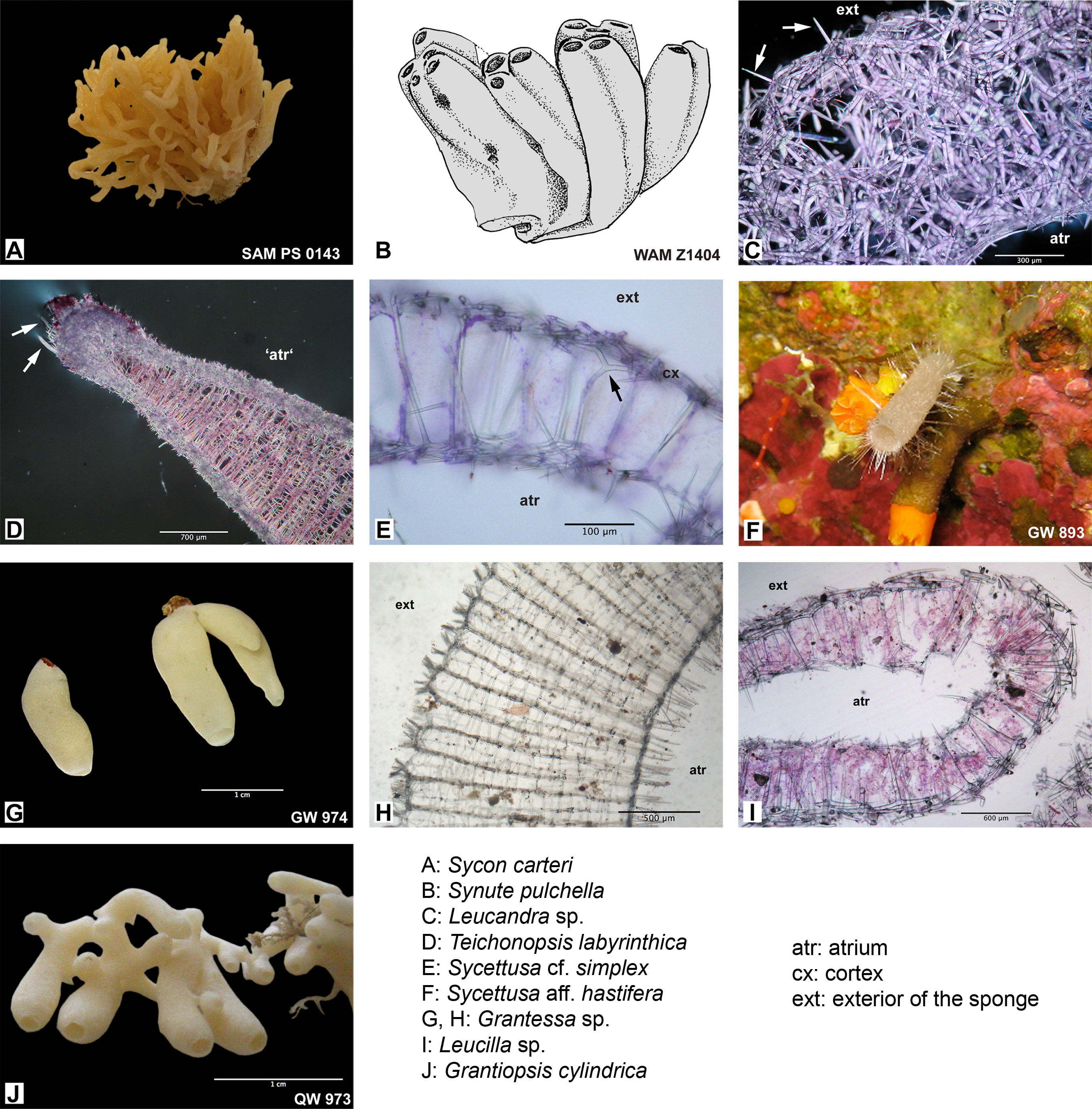

Supplement: Figure S2 — Included new specimens of Calcaronea (habitus and transversial sections). A: Sycon carteri (in phylogeny: SAM PS0142, a conspecific specimen); B: Synute pulchella (drawn from photography); D: Leucandra sp. (transversial section); D: Teichonopsis labyrinthica (transversial section). Due to the growth-form the upper surface corresponds to the atrium. E: Sycettusa cf. simplex (transversial section). The arrow points at the unpaired angle of a pseudosagittal triactine. F: Sycettusa aff. hastifera in-situ; G,H: Grantessa sp. GW974; I: Leucilla sp. (transversial section); J: Grantiopsis cylindrica. atr: atrium; cx: cortex; ext: exterior of the sponge. (TIF) [file pone.0033417.s002.tif]

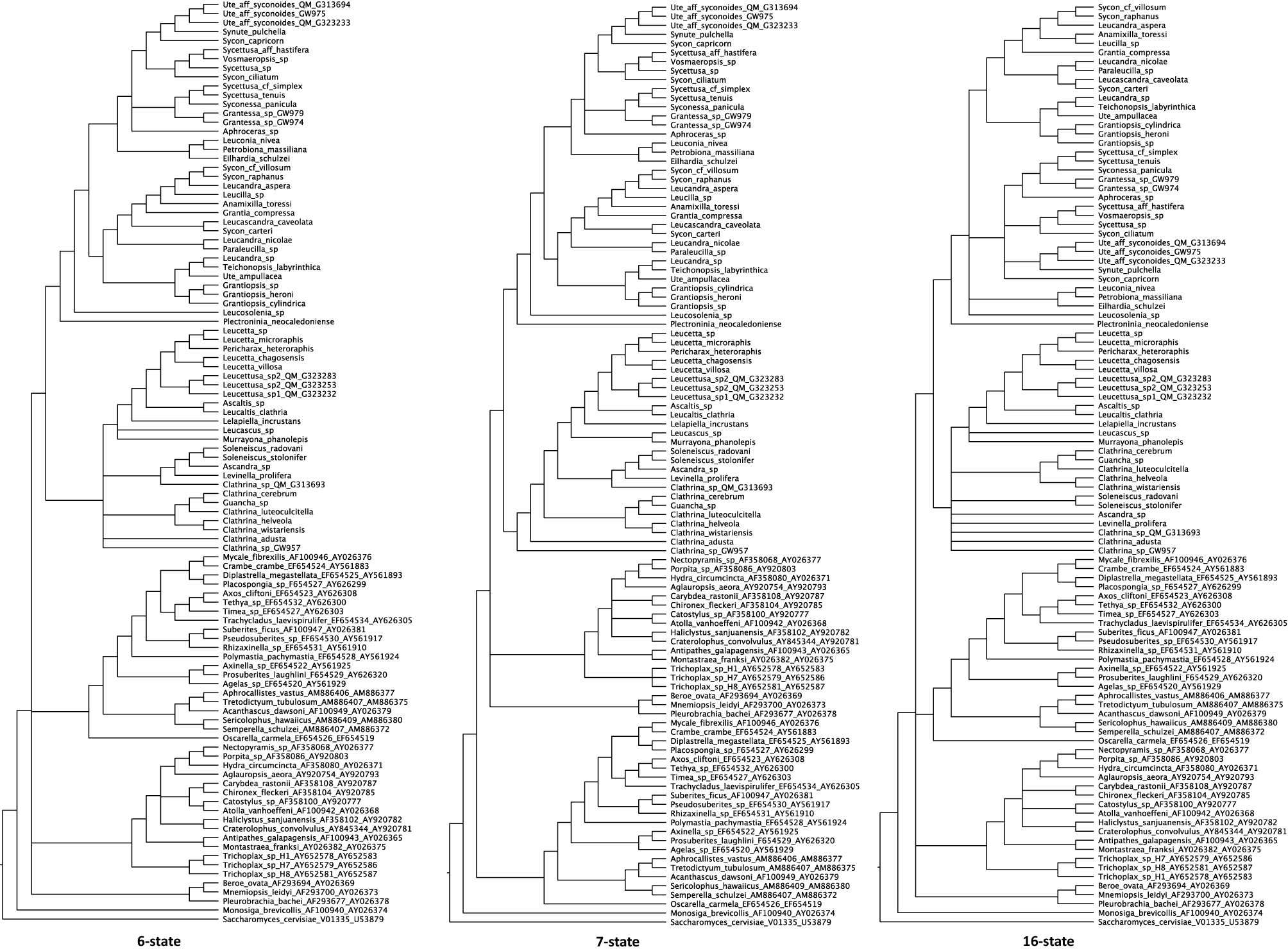

Supplement: Figure S4 — Strict consensus of Bayesian phylogenies obtained with different doublet models of each model family. 6-state: 6A–6F; 7-state: 7A–7F; 16-state: 16A–16F). Polytomies indicate model specific differences in tree topologies. (TIF) [file pone.0033417.s004.tif]

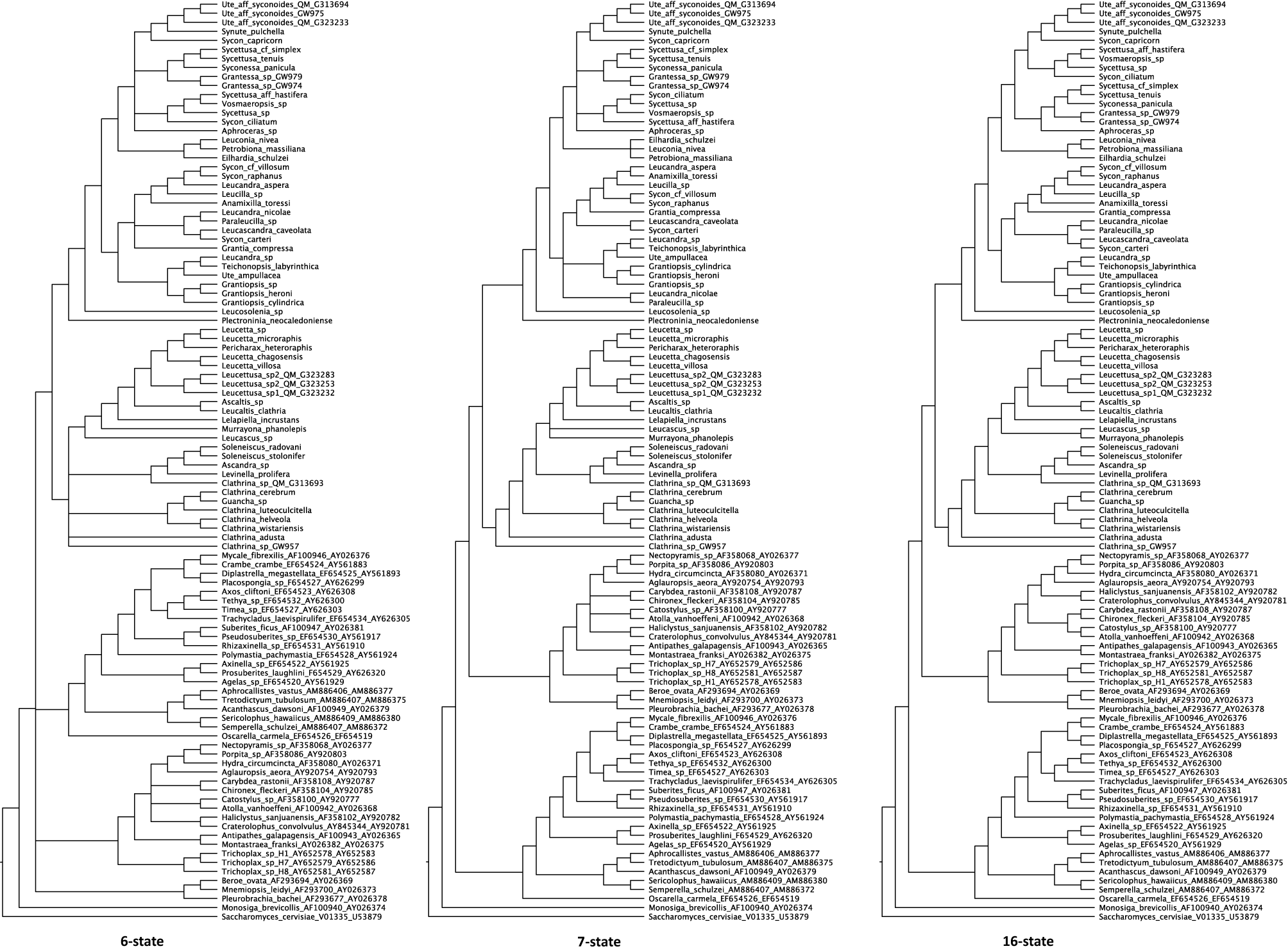

Supplement: Figure S5 — Strict consensus of ML phylogenies obtained with different doublet models of each model family. 6-state: 6A–6E; 7-state: 7A–7E; 16-state: 16A, 16B). Polytomies indicate model specific differences in tree topologies. (TIF) [file pone.0033417.s005.tif]

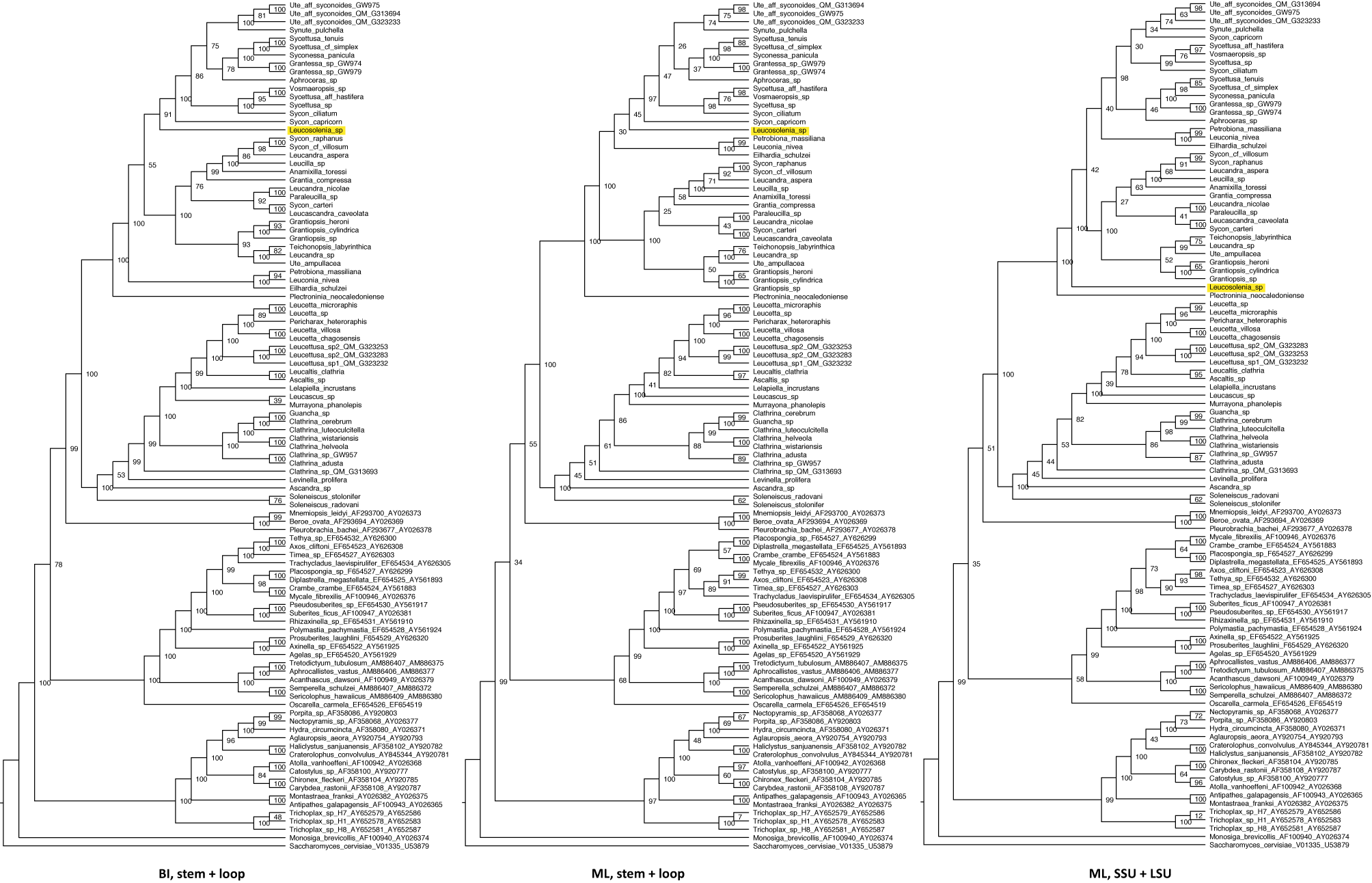

Supplement: Figure S6 — Phylogenies obtained under different partitioning schemes (stem+loop, SSU+LSU) with standard, non-doublet models. Support values (ML:BS, BI:PP) are given at the nodes. With a partitioning of stem+loop, the position of Leucosolenia differs from trees inferred with doublet model (Figures S4, S5), while with a partitioning into SSU+LSU, the position is as presented in Figure 3. Note also that all analyses result in a different topology at the base of Calcinea compared to our preferred doublet-inferred phylogenies (see main text). (TIF) [file pone.0033417.s006.tif]

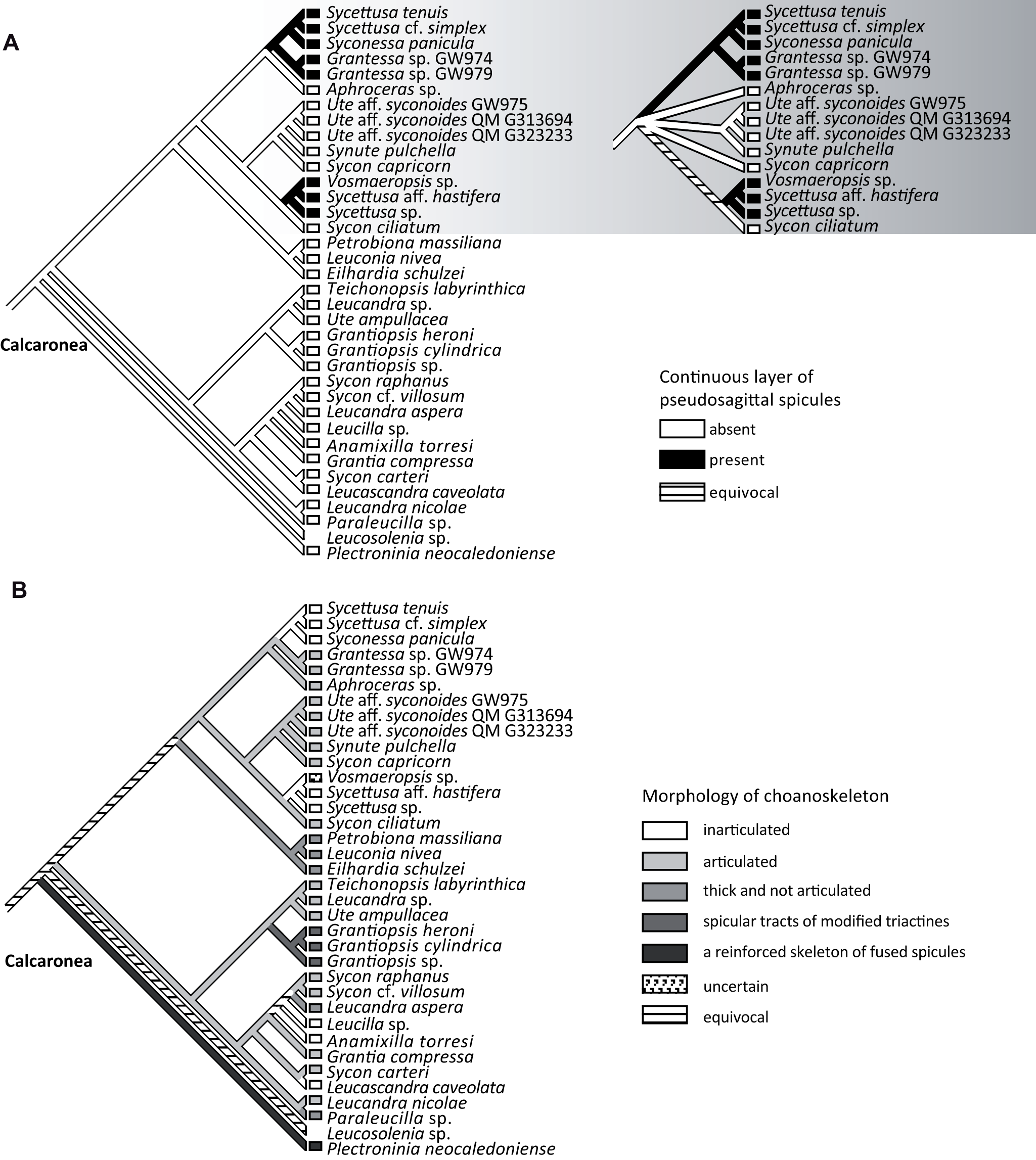

Supplement: Figure S7 — Evolution of pseudosagittal spicules and of the organization of the choanoskeleton. Tree topology identical to Fig.3, only class Calcaronea is shown. A. Presence of a continuous layer of pseudosagittal spicules. left: obtained phylogeny, right: Excerpt of clade LEUC I with nodes of PP-support below 90% collapsed. B. Morphology of the choanoskeleton (characters modified from [19]). Note that inarticulated choanoskeletons evolved at least four times from ancestors with articulated choanoskeletons. (TIF) [file pone.0033417.s007.tif]
