# Supplementary material for: Molecular Phylogenetic Evaluation of Classification and Scenarios of Character Evolution in Calcareous Sponges (Porifera, Class Calcarea)
Source: PLoS One. 2012 Mar 27;7(3):e33417. doi: 10.1371/journal.pone.0033417 (PMC3314023; doi:10.1371/journal.pone.0033417)
Supplement: Table S1 — LSU rRNA primer sequences. (PDF) [file pone.0033417.s008.pdf]

Table S1: LSU rRNA primer sequences

Name	Sequence (5'-3')	Reference
**F63mod**	ACCCGCTGAAYTTAAGCATATHANTMAG	[1]
**28S-350rv**	CTTTCCCTCACGGTACTTG	this study
**28S-560rv**	CTTTCAACGGYTTCACGTGC	this study
**28S-C2-fwd**	GAAAAGAACTTTGRARAGAGAGT	[2]
**28S-D2-rev**	TCCGTGTTTCAAGACGGG	[2]
**NL4F**	GACCCGAAAGATGGTGAACTA	[3]
**NL4R**	ACCTTGGAGACCTGATGCG	[3]
**28S-1260fw**	ATTCTCAAACTTTAAATBGGTAAG	this study
**28S-1340rv**	CATCGCCAGTTCTGCTTAC	this study
**28S-1810fw**	CGAAAGGGAATCGGGTTAATATTCC	this study
**28S-2490fw**	CAACCAAGCGCGGGTAAACG	this study
**28S-2570rv**	AATCTCGTTAATCCATTCATGC	this study
**28S-2634fw**	TCAAAGTGAAGAAATTCAACCAAGC	this study
**R3264**	TTCYGACTTAGAGGCGTTCAG	[1]

Additional references

1. Medina M, Collins AG, Silberman JD, Sogin ML (2001) Evaluating hypotheses of basal animal phylogeny using complete sequences of large and small subunit rRNA. Proc Natl Acad Sci USA 98: 9707-9712.
2. Chombard C, Boury-Esnault N, Tillier S (1998) Reassessment of homology of morphological characters in tetractinellid sponges based on molecular data. Syst Biol 47: 351-366.
3. Nichols SA (2005) An evaluation of support for order-level monophyly and interrelationships within the class Demospongiae using partial data from the large subunit rDNA and cytochrome oxidase subunit I. Mol Phylogenet Evol 34: 81-96.
