# Supplementary material for: Molecular Phylogenetic Evaluation of Classification and Scenarios of Character Evolution in Calcareous Sponges (Porifera, Class Calcarea)
Source: PLoS One. 2012 Mar 27;7(3):e33417. doi: 10.1371/journal.pone.0033417 (PMC3314023; doi:10.1371/journal.pone.0033417)
Supplement: Table S2 — GenBank accession numbers of outgroup taxa. (PDF) [file pone.0033417.s009.pdf]

Table S2: GenBank accession numbers of outgroup taxa

Species	GenBank accession	
	SSU	LSU
**Fungi**		
Saccharomyces cervisiae	V01335	U53879
**Choanoflagellata**		
Monosiga_brevicollis	AF100940	AY026374
**Porifera: Demospongiae**		
Agelas sp.	EF654520	AY561929
Axinella sp.	EF654522	AY561925
Axos cliftoni	EF654523	AY626308
Crambe crambe	EF654524	AY561883
Diplastrella megastellata	EF654525	AY561893
Mycale fibrexilis	AF100946	AY026376
Placospongia sp.	F654527	AY626299
Polymastia pachymastia	EF654528	AY561924
Prosuberites laughlini	F654529	AY626320
Pseudosuberites sp	EF654530	AY561917
Rhizaxinella sp.	EF654531	AY561910
Suberites ficus	AF100947	AY026381
Tethya sp.	EF654532	AY626300
Timea sp.	EF654527	AY626303
Trachycladus laevispirulifer	EF654534	AY626305
**Porifera: Hexactinellida**		
Acanthascus dawsoni	AF100949	AY026379
Aphrocallistes vastus	AM886406	AM886377
Sericolophus hawaiicus	AM886409	AM886380
Semperella schulzei	AM886407	AM886372
Tretodictyum tubulosum	AM886407	AM886375
**Porifera: Homoscleromorpha**		
Oscarella carmela	EF654526	EF654519
**Cnidaria: Anthozoa**		
Antipathes galapagensis	AF100943	AY026365
Montastraea franksi	AY026382	AY026375
**Cnidaria: Cubozoa**		
Carybdea rastonii	AF358108	AY920787
Chironex fleckeri	AF358104	AY920785
**Cnidaria: Hydrozoa**		
Aglauropsis aeora	AY920754	AY920793
Hydra circumcincta	AF358080	AY026371
Nectopyramis sp	AF358068	AY026377
Porpita sp	AF358086	AY920803
**Cnidaria: Scyphozoa**		
Atolla vanhoeffeni	AF100942	AY026368
Catostylus sp	AF358100	AY920777

Species	GenBank accession	
	SSU	LSU
**Cnidaria: Staurozoa**		
Craterolophus convolvulus	AY845344	AY920781
Haliclystus sanjuanensis	AF358102	AY920782
**Ctenophora**		
Pleurobrachia bachei	AF293677	AY026378
Beroe ovata	AF293694	AY026369
Mnemiopsis leidyi	AF293700	AY026373
**Placozoa**		
Trichoplax sp. H1	AY652578	AY652583
Trichoplax sp. H7	AY652579	AY652586
Trichoplax sp. H8	AY652581	AY652587
