# Supplementary material for: Molecular Phylogenetic Evaluation of Classification and Scenarios of Character Evolution in Calcareous Sponges (Porifera, Class Calcarea)
Source: PLoS One. 2012 Mar 27;7(3):e33417. doi: 10.1371/journal.pone.0033417 (PMC3314023; doi:10.1371/journal.pone.0033417)
Supplement: Figure S3 — LSU secondary structure ( Leucetta microraphis ). (PDF) [file pone.0033417.s003.pdf]

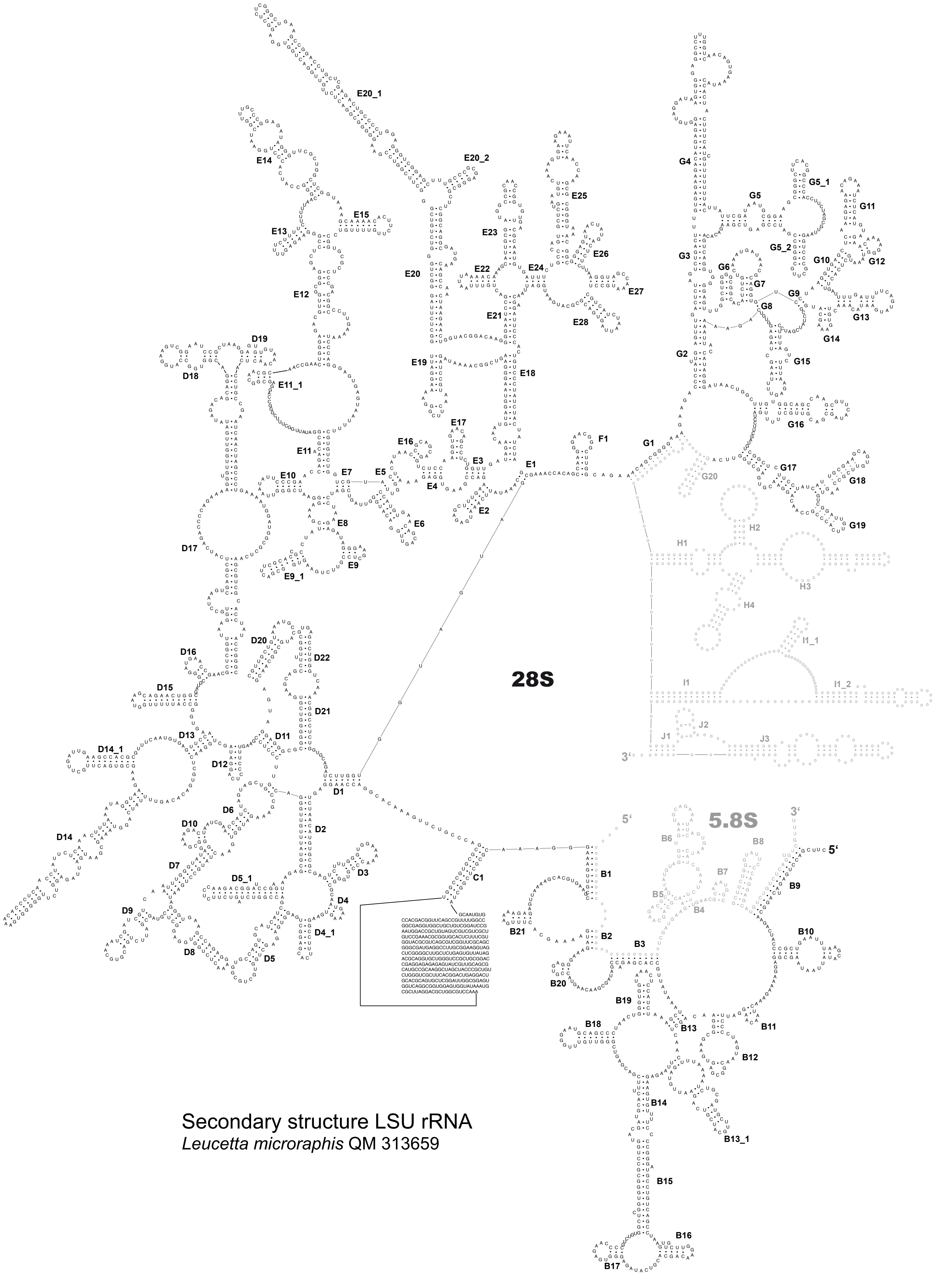

Secondary structure LSU rRNA
Leucetta microraphis QM 313659
